# Planning horizon affects prophylactic decision-making and epidemic dynamics

**DOI:** 10.7717/peerj.2678

**Published:** 2016-11-08

**Authors:** Luis G. Nardin, Craig R. Miller, Benjamin J. Ridenhour, Stephen M. Krone, Paul Joyce, Bert O. Baumgaertner

**Affiliations:** 1 Center for Modeling Complex Interactions, University of Idaho, Moscow, ID, United States; 2 Department of Biological Sciences, University of Idaho, Moscow, ID, United States; 3 Department of Mathematics, University of Idaho, Moscow, ID, United States; 4 Department of Politics and Philosophy, University of Idaho, Moscow, ID, United States

**Keywords:** Human behavior, Behavioral epidemiology, ODE, Infectious diseases, Prophylaxis, Agent-based modeling

## Abstract

The spread of infectious diseases can be impacted by human behavior, and behavioral decisions often depend implicitly on a planning horizon—the time in the future over which options are weighed. We investigate the effects of planning horizons on epidemic dynamics. We developed an epidemiological agent-based model (along with an ODE analog) to explore the decision-making of self-interested individuals on adopting prophylactic behavior. The decision-making process incorporates prophylaxis efficacy and disease prevalence with the individuals’ payoffs and planning horizon. Our results show that for short and long planning horizons individuals do not consider engaging in prophylactic behavior. In contrast, individuals adopt prophylactic behavior when considering intermediate planning horizons. Such adoption, however, is not always monotonically associated with the prevalence of the disease, depending on the perceived protection efficacy and the disease parameters. Adoption of prophylactic behavior reduces the epidemic peak size while prolonging the epidemic and potentially generates secondary waves of infection. These effects can be made stronger by increasing the behavioral decision frequency or distorting an individual’s perceived risk of infection.

## Introduction

Human behavior plays a significant role in the dynamics of infectious disease (Ferguson, 2007; Funk, Salathé & Jansen, 2010). However, the inclusion of behavior in epidemiological modeling introduces numerous complications and involves fields of research outside the biological sciences, including psychology, philosophy, sociology, and economics. Areas of research that incorporate human behavior into epidemiological models are loosely referred to as *social epidemiology*, *behavioral epidemiology*, or *economic epidemiology* (Manfredi & D’Onofrio, 2013; Philipson, 2000). We use the term ‘behavioral epidemiology’ to broadly refer to all epidemiological approaches that incorporate human behavior. While the incorporation of behavior faces many challenges (Funk et al., 2015), one of the goals of behavioral epidemiology is to understand how social and behavioral factors affect the dynamics of infectious disease epidemics. This goal is usually accomplished by coupling models of social behavior and decision making with biological models of contagion (Perrings et al., 2014; Funk, Salathé & Jansen, 2010).

Many social and behavioral aspects can be incorporated into a model of infectious disease. One example is the effect of either awareness or fear spreading through a population (Funk et al., 2009; Epstein et al., 2008). In these types of models, the spread of beliefs or information is treated as a contagion much like an infectious disease, though the network for the spread of information may differ from the biological network (Bauch & Galvani, 2013). Other models focus on how individuals adapt their behavior by weighting the risk of infection with the cost of social distancing (Fenichel et al., 2011; Reluga, 2010) or other disincentives (Auld, 2003). Still others model public health interventions (e.g., isolation, vaccination, surveillance, etc.) and individual responses to them (Del Valle et al., 2005). Many of these models sit at the population level, incorporating the effects of social factors and abstracting away details about the individuals themselves.

The SPIR model (**S**usceptible, **P**rophylactic, **I**nfectious, **R**ecovered) developed here, in contrast, is an epidemiological model that couples individual behavioral decisions with an extension of the SIR model (Kermack & McKendrick, 1927). In this model agents that are vulnerable to infection may be in one of two states, *susceptible* or *prophylactic*, which is determined by their behavior. Agents in the susceptible state engage in the status quo behavior while agents in the prophylactic state employ preventative behaviors that reduce their chance of infection. We use a rational choice model to represent individual behavioral decisions, where individuals select the largest utility between engaging in prophylactic behavior (e.g., hand-washing or wearing a face mask) or non-prophylactic behavior (akin to the status quo). We also allow for the fact that individuals may not perceive the risk of getting infected accurately, but rather receive distorted information, for example, through the media.

We use the SPIR model to understand how an individual’s planning horizon—the time in the future over which individuals calculate their utilities to make a behavioral decision—affects behavioral change and how that, in turn, influences the dynamics of an epidemic.

One of our key findings is that individuals choose to engage in prophylactic behavior only when the planning horizon is “just right.” If the planning horizon is set too far into the future, it is in an individual’s best interest to become infected (i.e., get it over with); if the horizon is too short, individuals dismiss the future risk of infection (i.e., live for the moment). What counts as “just right”, too short, and too far (i.e., the time scale of the planning horizon) depend on the disease in question. We explore two hypothetical contrasting diseases, one with long recovery time and acute severity, and another with short recovery time and mild severity.

## Methods

### The SPIR model

The SPIR model couples two sub-models: one, the *Disease Dynamics Model*, reproducing the dynamics of the infectious disease, and another, the *Behavioral Decision Model*, that determines how agents in the disease dynamics model make the decision to engage in prophylactic or non-prophylactic behavior.

#### Disease dynamics model

**Figure 1:**
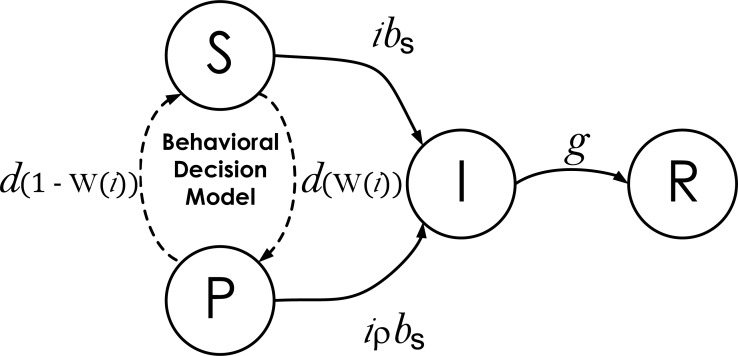
State-transition diagram of agents in the Disease Dynamics Model. S, P, I, and R represent the four epidemiological states an agent can be in: Susceptible, Prophylactic, Infectious, and Recovered, respectively. The parameters over the transitions connecting the states represent the probability per time step that agents in one state move to an adjacent state: *i* is the proportion of infectious agents in the population; *b*_S_ and *b*_P_ are the respective probabilities that an agent in state S or P, encountering an infectious agent I, becomes infected; *g* is the recovery probability; *ρ* is the reduction in the transmission probability when an agent adopts prophylactic behavior that linearly relates *b*_S_ and *b*_P_, i.e., *b*_P_ = *ρb*_S_; *d* is the behavioral decision making probability; and W(*i*) is an indicator function returning value 1 when the utility of being prophylactic is greater than the utility of being susceptible and 0 otherwise (see details in Behavioral Decision Model).

The disease dynamics model reproduces the dynamics of the infectious disease in a constant population of *N* agents. Each agent can be in one of four states: Susceptible (S), Prophylactic (P), Infectious (I), or Recovered (R). The difference between agents in states S and P is that the former engage in non-prophylactic behavior and do not implement any measure to prevent infection, while the latter adopt prophylactic behavior which decreases their probability of being infected (e.g., wearing a mask, washing hands, etc.). The adoption of prophylactic behavior, however, imposes costs on individuals that may prevent them from engaging in such behavior. In Western countries, for example, wearing a mask, other than uncomfortable, may signal a lack of trust in others or an unhealthy state, thus reducing social contacts. Agents in the infectious state I are infected and infective, while those in the recovered state R are immune to and do not transmit disease. The transition between states is captured with the state-transition diagram shown in Fig. 1. For reference, all the parameters and variables in the SPIR model are listed and defined in Table 1.

**Table 1:** Parameters and state variables of the SPIR model.

Symbol	Definition
*N*	Total number of agents in the population.
S	An agent in the susceptible state engaging in non-prophylactic behavior.
P	An agent in the prophylactic state engaging in prophylactic behavior.
I	An agent in the infectious state.
R	An agent in the recovered state.
*s*	Proportion of susceptible agents in the population.
*p*	Proportion of prophylactic agents in the population.
*i*	Proportion of infectious agents in the population.
*r*	Proportion of recovered agents in the population.
*b*_S_	Probability that an agent in the susceptible state becomes infected upon interacting with an infectious agent.
*b*_P_	Probability that an agent in the prophylactic state becomes infected upon interacting with an infectious agent.
*ρ*	Reduction in the transmission probability or rate when adopting prophylactic behavior: *b*_P_ = *ρb*_S_ (0 ≤ *ρ* ≤ 1). Note that we refer to 1 − *ρ* as the *protection*.
*g*	Probability an infectious agent recovers.
*d*	Probability an agent in the susceptible or prophylactic state decides which behavior to engage in.
*κ*	Distortion of the perceived proportion of infectious agents in the population (i.e., *distortion factor*).
*u*_Y_	Payoff per time step for being in state Y, where Y ∈ {S, P, I, R}. Note that (*u*_S_ − *u*_P_) is the cost of adopting prophylactic behavior.
$\text{E} \left[ {T}_{\text{Y}{|}\text{}{\text{D}}_{\text{X}}\text{}} \right] $	Number of time steps agents expect to spend in state Y assuming they decide to adopt state X (i.e., D_X_), where Y ∈ {S, P, I, R} and X ∈ {S, P}.
U_X_	Utility for adopting state X, where X ∈ {S, P}.
H	Time into the future at which agents calculate their current utilities (i.e., *planning horizon*).
$\text{W} \left( i \right) $	Indicator function returning value 1 when the utility of adopting prophylactic behavior is greater than the utility of adopting non-prophylactic behavior and 0 otherwise.

This sub-model assumes that in each time step, three types of events occur: (1) interactions among agents and any infections that may result, (2) behavioral decisions to engage in prophylactic or non-prophylactic behavior, and (3) recoveries. All agents interact by pairing themselves with another randomly selected agent in the population. Given four possible states, there are ten possible pairwise interactions. However, only two types of interactions can change the state of an agent: 〈S, I〉 and 〈P, I〉. For the interaction 〈S, I〉, the susceptible agent S is infected by the infectious agent I with probability *b*_S_. For the interaction 〈P, I〉, the prophylactic agent P is infected by the infectious agent I with probability *b*_P_, where *b*_P_ ≤ *b*_S_. The probability *b*_P_ is linearly related to probability *b*_S_ by the coefficient *ρ* (i.e., *b*_P_ = *ρb*_S_). Thus (1 − *ρ*) is the protection acquired by adopting prophylactic behavior. Assuming well-mixed interactions, the proportion of infectious agents *i* represents the probability that an agent is paired with an infectious agent and the per time step probability of an agent in state S or P being infected is *ib*_S_ or *ib*_P_, respectively. In addition to interacting, susceptible and prophylactic agents have probability *d* per time step of making a behavioral decision to engage in prophylactic or non-prophylactic behavior. The agents’ behavioral decision is reflected in the indicator function W(*i*) (see details in Behavior Decision Model), and they engage in the prophylactic behavior when W(*i*) = 1 (i.e., adopt state P) and the non-prophylactic behavior when W(*i*) = 0 (i.e., adopt state S). Infectious agents have probability *g* per time step of recovering. We implemented an agent-based version of the disease dynamics model using the Gillespie algorithm (Gillespie, 1976) (source code available in Supplemental Information 1).

Our agent-based model (ABM) is a useful tool for exploring the aggregated effects of individual decision making, including scenarios where populations are heterogeneous (e.g., some individuals may be more risk-averse than others). A drawback of this model is that it requires simulations that can be computationally intensive. The stochasticity of the ABM further exacerbates the computational burden when mean results are desired. If, however, we assume that the population is well-mixed, the dynamics can be generated using a system of ODEs (see Sec. S7 in Supplemental Information 1 for a comparison):

(1a) \begin{eqnarray*} \frac{ds}{dt} =-\beta si-\delta s\text{W}(i)+\delta p \left( 1-\text{W}(i) \right) \end{eqnarray*}

(1b) \begin{eqnarray*} \frac{dp}{dt} =-\rho \beta pi+\delta s\text{W}(i)-\delta p \left( 1-\text{W}(i) \right) \end{eqnarray*}

(1c) \begin{eqnarray*} \frac{di}{dt} =\beta si+\rho \beta pi-\gamma i\end{eqnarray*}

(1d) \begin{eqnarray*} \frac{dr}{dt} =\gamma i\end{eqnarray*}

where *s*, *p*, *i*, and *r* are the proportion of susceptible, prophylactic, infectious, and recovered agents in the population. The parameters *β*, *γ*, and *δ* are transmission, recovery, and decision rates, whose equivalent probabilities are, respectively, transmission (*b*_S_), recovery (*g*), and decision (*d*) (Table 2). The parameter *ρ* refers to the reduction in transmission rate when adopting prophylactic behavior. We convert between rates and probabilities using equations $x=-\ln \left( 1-y \right) $ and *y* = 1 − *e*^−*x*^, where *x* and *y* are rate and probability values respectively (Fleurence & Hollenbeak, 2007). One unit of continuous time in the ODE model corresponds to *N* time steps in the ABM.

**Table 2:** Disease parameters in rates and probabilities.

Rate parameter	Probability parameter	Description	Probability to rate conversion
*β*	*b*_S_	Transmission	$\beta =-\ln \left( 1-{b}_{\text{S}} \right) $
*γ*	*g*	Recovery	$\gamma =-\ln \left( 1-g \right) $
*δ*	*d*	Decision	$\delta =-\ln \left( 1-d \right) $

#### Behavioral decision model

Recall that agents have per step probability *d* of making a behavioral decision; here we specify how those decisions are made. The behavioral decision model, in principle, can be any model that enables agents to decide whether or not to engage in prophylactic behavior. Our decision model is a rational choice model that assumes agents are self-interested and rational; thus they adopt the behavior with the largest utility over the planning horizon, H. Note that the planning horizon is a construct used to calculate utilities and it does not affect the time until an agent has an opportunity to make another decision within the disease dynamics model.

The planning horizon is the time in the future over which agents calculate their utilities. In order for the agent to make these calculations, we make the following assumptions about agents tasked with making a decision; (1) Agents have identical and complete knowledge of the relevant disease parameters, *b*_S_, *b*_P_, and *g*. (2) Prophylactic behavior has the same protection efficacy, *ρ*, for all agents; (3) Agents believe the current prevalence of the disease is *i* (in the case of no distortion—see below); (4) Agents assume that *i* will remain at its current value during the next H time steps; (5) Agents compute expected waiting times based on a censored geometric distribution. Specifically, they believe that they will spend the amount of time *T*_X_ in state X ∈ {S, P}, where the time *T*_X_ has a geometric distribution with parameter *ib*_X_, censored at the planning horizon H. When their time in X is over, agents know they will move to state I where the amount of time they expect to spend in state I has a geometric distribution with parameter *g* censored at the time remaining, H − *T*_X_. When their time in state I is over, they know they will move to state R where they will remain until time H. (6) Agents know the per time step payoff for each state *u*_Y_, where Y ∈ {S, P, I, R}, and all agents are assumed to have the same set of payoff values. (7) Agents calculate the sum of the utility from now until time H under the two possible behavioral decisions (D_S_ or D_P_).

*Perfect knowledge of i.* To calculate the utilities when they have perfect knowledge of *i*, agents use the length of time they expect to spend in each state. We begin by deriving the expected time in state X, where X ∈ {S, P}, and then we use this result to derive the expected time in I and, finally, R. The expected time in state I is conditioned on *T*_X_ because agents are rational and they average the time they expect to spend on state I over all possible hypothetical combinations involving X and I up to H. To simplify notation, it is helpful to first define the force of infection for state X, *f*_X_: *f*_S_ = *ib*_S_ and *f*_P_ = *iρb*_S_. For the agent considering hypothetical futures, the planning horizon serves to censor all waiting times greater than H, giving them value H. This leads to the following probability mass function for the time spent in state X should they decide on behavior X (denoted *T*_X|D_X__),

(2) \begin{eqnarray*}\Pr \left( {T}_{\text{X}{|}\text{}{\text{D}}_{\text{X}}\text{}}=t \right) = \left\{ \begin{array}{@{}ll@{}} \displaystyle {f}_{\text{X}}{ \left( 1-{f}_{\text{X}} \right) }^{t},\hspace*{10.00002pt}&\displaystyle \hspace*{10.00002pt}0\leq t\lt \text{H}\cvskip[5pt]\\ \displaystyle { \left( 1-{f}_{\text{X}} \right) }^{\text{H}},\hspace*{10.00002pt}&\displaystyle \hspace*{10.00002pt}t=\text{H}\cvskip[5pt]\\ \displaystyle 0,\hspace*{10.00002pt}&\displaystyle \hspace*{20.00003pt}\text{otherwise}. \end{array} \right. \end{eqnarray*}

The expected time spent in state X can be expressed as

(3) \begin{eqnarray*}\text{E} \left[ {T}_{\text{X}{|}\text{}{\text{D}}_{\text{X}}\text{}} \right] =\sum _{t=0}^{\text{H}}t{f}_{\text{X}}{ \left( 1-{f}_{\text{X}} \right) }^{t}+\text{H}{ \left( 1-{f}_{\text{X}} \right) }^{\text{H}},\end{eqnarray*}

which simplifies to the desired expectation,

(4) \begin{eqnarray*}\text{E} \left[ {T}_{\text{X}{|}\text{}{\text{D}}_{\text{X}}\text{}} \right] = \left( \frac{1}{{f}_{\text{X}}} -1 \right) \left( 1-{ \left( 1-{f}_{\text{X}} \right) }^{\text{H}} \right) .\end{eqnarray*}

Notice that $ \left( \frac{1}{{f}_{\text{X}}} -1 \right) $ is the expected time to remain in state X before moving to state I for an uncensored geometric with minimum value of zero, while the second parenthetical term rescales the expectation to the interval [1, H].

Next, we derive the expected time spent in I by conditioning on *T*_X|D_X__.

(5) \begin{eqnarray*}\text{E} \left[ {T}_{\text{I}{|}\text{}{\text{D}}_{\text{X}}\text{}} \right] =\text{E} \left[ \text{E} \left[ {T}_{\text{I}{|}\text{}{\text{D}}_{\text{X}}\text{}}{|}{T}_{\text{X}{|}\text{}{\text{D}}_{\text{X}}\text{}} \right] \right] =\text{E} \left[ \left( \frac{1}{g} -1 \right) \left( 1-{ \left( 1-g \right) }^{\text{H}-{T}_{\text{X}{|}\text{}{\text{D}}_{\text{X}}\text{}}} \right) \right] .\end{eqnarray*}

After considerable algebra (see Sec. S3 in Supplemental Information 1), we get the expectation,

(6) \begin{eqnarray*}\text{E} \left[ {T}_{\text{I}{|}\text{}{\text{D}}_{\text{X}}\text{}} \right] = \left( \frac{1}{g} -1 \right) \left( \frac{ \left( \frac{1}{g} -1 \right) \left( 1-{ \left( 1-g \right) }^{\text{H}} \right) - \left( \frac{1}{{f}_{\text{X}}} -1 \right) \left( 1-{ \left( 1-{f}_{\text{X}} \right) }^{\text{H}} \right) }{ \left( \frac{1}{g} -1 \right) - \left( \frac{1}{{f}_{\text{X}}} -1 \right) } \right) .\end{eqnarray*}

Again, the expectation of the uncensored geometric is $ \left( \frac{1}{g} -1 \right) $. The second parenthetical term compresses the expected time into the interval between $\text{E} \left[ {T}_{\text{X}{|}\text{}{\text{D}}_{\text{X}}\text{}} \right] $ and H. Notice that Eq. (6) is defined only so long as *f*_X_ ≠ *g*. When *f*_X_ = *g*, we instead have

(7) \begin{eqnarray*}\text{E} \left[ {T}_{\text{I}{|}\text{}{\text{D}}_{\text{X}}\text{}} \right] = \left( \frac{1}{g} -1 \right) \left( 1-{ \left( 1-g \right) }^{\text{H}} \right) -\text{H}{ \left( 1-g \right) }^{\text{H}+1}.\end{eqnarray*}

Finally, the agent calculates the expected time in state R by subtracting the expectations for X and I from H,

(8) \begin{eqnarray*}\text{E}[{T}_{\text{R}{|}\text{}{\text{D}}_{\text{X}}\text{}}]=\text{H}-\text{E} \left[ {T}_{\text{X}{|}\text{}{\text{D}}_{\text{X}}\text{}} \right] -\text{E} \left[ {T}_{\text{I}{|}\text{}{\text{D}}_{\text{X}}\text{}} \right] .\end{eqnarray*}

Notice that for each of the expected waiting times calculated in Eqs. (4), (6) and (7), as H goes to infinity, the rescaling terms go to one so that the equations yield the familiar expected values for the uncensored geometrics.

**Table 3:** Input parameters for two hypothetical contrasting diseases.

Parameter		Disease 1	Disease 2
Type	Name		
Biological	R_0_	2	2
	*β*	0.031	0.25
	*ρ*	0.1	0.01
	*γ*	0.015	0.125
	Recovery Time (1∕*γ*)	65	8
Behavioral	*δ*	0	0
	*κ*	1	1
	{*u*_S_, *u*_P_, *u*_I_, *u*_R_}	{1, 0.95, 0.1, 0.95}	{1, 0.95, 0.6, 1}

Having calculated these expected waiting times, the agent then calculates the utility for the two possible behaviors using,

(9) \begin{eqnarray*}{\text{U}}_{\text{S}}={u}_{\text{S}}\text{E} \left[ {\text{}T\text{}}_{\text{S}{|}\text{}{\text{D}}_{\text{S}}\text{}} \right] +{u}_{\text{I}}\text{E} \left[ {\text{}T\text{}}_{\text{I}{|}\text{}{\text{D}}_{\text{S}}\text{}} \right] +{u}_{\text{R}}\text{E} \left[ {\text{}T\text{}}_{\text{R}{|}\text{}{\text{D}}_{\text{S}}\text{}} \right] \end{eqnarray*}

and

(10) \begin{eqnarray*}{\text{U}}_{\text{P}}={u}_{\text{P}}\text{E} \left[ {\text{}T\text{}}_{\text{P}{|}\text{}{\text{D}}_{\text{P}}\text{}} \right] +{u}_{\text{I}}\text{E} \left[ {\text{}T\text{}}_{\text{I}{|}\text{}{\text{D}}_{\text{P}}\text{}} \right] +{u}_{\text{R}}\text{E} \left[ {\text{}T\text{}}_{\text{R}{|}\text{}{\text{D}}_{\text{P}}\text{}} \right] .\end{eqnarray*}

Note that when agents calculate expected times for states S and P, they need not consider the possibility of alternating to the other state in the future. This is because they assume a constant *i* which implies the best strategy now will remain the best strategy at all times during H. Thus, $\text{E} \left[ {\text{}T\text{}}_{\text{S}{|}\text{}{\text{D}}_{\text{P}}\text{}} \right] $ and $\text{E} \left[ {\text{}T\text{}}_{\text{P}{|}\text{}{\text{D}}_{\text{S}}\text{}} \right] $ are both zero. To be clear, this constraint pertains only to calculating utilities; agents are not constrained in how many times they actually switch states during the epidemic.

Because decisions reflect the largest utility and because the population is homogeneous, the behavioral decision can be expressed as the indicator function $\text{W} \left( i \right) $ defined by

(11) \begin{eqnarray*}\text{W}(i)= \left\{ \begin{array}{@{}ll@{}} \displaystyle 1\hspace*{10.00002pt}&\displaystyle \hspace*{20.00003pt}\text{for}{\text{U}}_{\text{S}}\lt {\text{U}}_{\text{P}}\\ \displaystyle 0\hspace*{10.00002pt}&\displaystyle \hspace*{20.00003pt}\text{otherwise} \end{array} \right. \end{eqnarray*}

*Distorting knowledge of i*. Recall that assumption (3) that underlies the behavioral decision model is that agents know the prevalence of the disease accurately. We relax this assumption to investigate how distorting this information affects the epidemic dynamics. To achieve this, we replace *i* with ${i}^{ \frac{1}{\kappa } }$ in the calculation of utilities where *κ* serves as a *distortion factor*. When *κ* = 1, *i* is not distorted; when *κ* > 1, the agent perceives *i* to be above its real value and when *κ* < 1 the opposite is true. To implement this distortion, we redefine *f*_X_ in the expected waiting time equations (i.e., Eqs. (3)–(7)) with ${f}_{\text{X}}={i}^{ \frac{1}{\kappa } }{b}_{\text{S}}$ when X = S and ${f}_{\text{X}}={i}^{ \frac{1}{\kappa } }\rho {b}_{\text{S}}$ when X = P.

## Results and Analysis

The SPIR model can be applied to a diverse range of infectious disease epidemics and how they might be impacted by human behavior. To illustrate specific characteristics of the model, however, we focus here on two contrasting diseases characterized by their severity, recovery time, and harm: *Disease 1* is acute, has a long recovery time, and may cause chronic harm, and *Disease 2* is mild, has a short recovery time, and causes no lasting harm. Table 3 shows the biological and behavioral parameter values used to generate the results discussed next, unless stated otherwise. Note that the results were generated using the ODE model that is more computationally efficient than the ABM, but generates the same results (see Sec. S7 in Supplemental Information 1 for a comparison).

### Behavioral decision analysis

**Figure 2:**
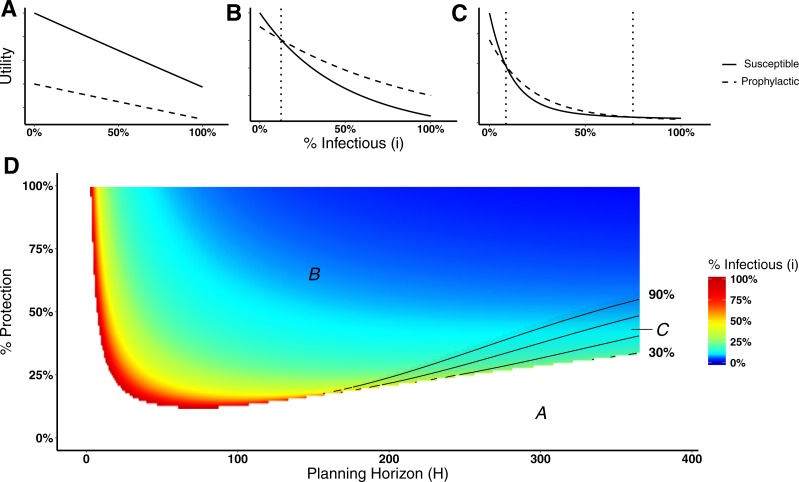
Heat map of switching points for Disease 1. (A) Situation in which non-prophylactic behavior is always more advantageous than prophylactic behavior regardless of the proportion of infectious agents. (B) Situation in which above a certain proportion of infectious agents (indicated by the vertical dotted line), the prophylactic behavior is more advantageous than non-prophylactic behavior. (C) Situation in which prophylactic behavior is more advantageous whenever the proportion of infectious agents is within a range of values represented by the two vertical dotted lines and less advantageous otherwise. (D) Proportion of infectious agents above which prophylactic behavior is more advantageous than non-prophylactic behavior given the percentage of protection (% Protection) obtained for adopting prophylactic behavior (1 − *ρ*) × 100 (*y*-axis) and the planning horizon H (*x*-axis). The three regions in (D) represent the situations shown in (A), (B), and (C). In region *A*, agents never adopt prophylactic behavior. In region *B*, agents adopt prophylactic behavior above the reported proportion of infectious agents. In region *C*, agents adopt prophylactic behavior only if the proportion of infected agents is between the proportion of infectious agents represented by the color gradient and the proportion represented by the contour lines.

Here we analyze the behavioral decision model used by the agents to decide whether or not to engage in prophylactic behavior. In particular, we are interested in identifying the level of disease prevalence above which agents would switch behavior, i.e., a *switching point*. A switching point is defined as the proportion of infectious agents beyond which it would be advantageous for an agent to switch from non-prophylactic to prophylactic behavior or vice-versa. We can visualize switching points by plotting the expected utility for the susceptible and prophylactic states as a function of the proportion of infectious agents. A switching point is where the expected utilities cross, if they cross. Figure 2A illustrates the situation in which the utilities do not cross, thus there is no switching point. Figure 2B illustrates the situation in which there is a single switching point; below the switching point the susceptible state has the higher utility, whereas above that point the prophylactic state has the higher utility. Figure 2C shows the situation in which the utilities cross twice, thus there are two switching points; between the switching points the prophylactic state has the higher utility, whereas the susceptible state has higher utility on the margins.

**Figure 3:**
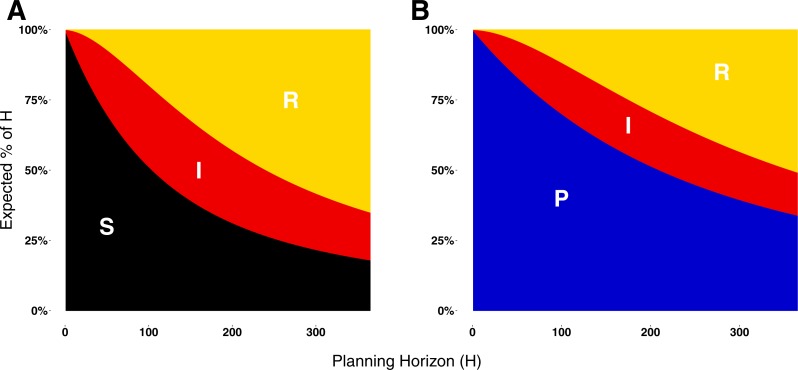
Expected proportion of the planning horizon spent in each state. (A) Proportion of the planning horizon agents expect to spend in each state if they decide to adopt state S. (B) Proportion of the planning horizon agents expect to spend in each state if they decide to adopt state P. For short planning horizons, the largest proportion of time is expected to be spent in the susceptible or prophylactic states. Increasing the planning horizon increases the proportion of H that an agent expects to spend in the recovered state.

Figure 2D shows a heat map of switching points for Disease 1 (see Fig. S1 for a heat map for Disease 2). The figure is divided into three regions—*A*, *B*, and *C*—that correspond to the three different utility situations illustrated in Figs. 2A, 2B, and 2C respectively. Region *A* corresponds to the situation in which agents never engage in prophylactic behavior because the utility of being in the susceptible state is never less than the prophylactic state regardless of disease prevalence (Fig. 2A). This situation occurs for low protection efficacy or short planning horizons. In the case of low protection efficacy, agents do not have an incentive to adopt prophylactic behavior because they expect to get infected regardless of their behavior. Thus, their best strategy is to become infected and then recover in order to collect the recovered payoff as quickly as possible (i.e., “get it over with”). In the case of short planning horizons, the relative contributions of the expected times of being in the susceptible or prophylactic state dominate the utility calculations, as shown in Fig. 3. The figure illustrates how, when the planning horizon is short, the expected percentage of time spent in the susceptible or prophylactic states are much greater than the expected percentage of time spent in the infectious or recovered state. Given that the susceptible payoff is greater than the prophylactic payoff, agents never adopt prophylactic behavior. The figure also shows how increasing the planning horizon changes the distribution of time spent in each state, which reduces the influence of the difference between the susceptible and prophylactic payoffs on behavioral decision.

**Figure 4:**
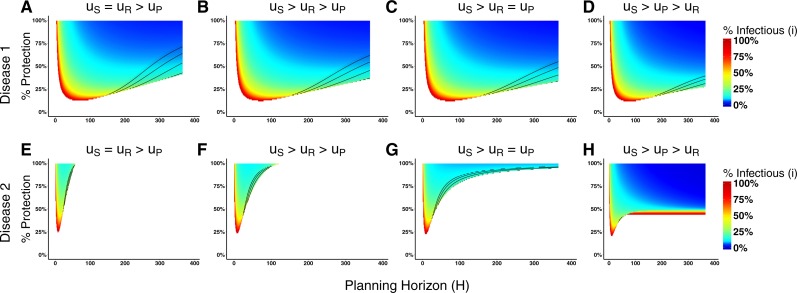
Heat maps of switching points under qualitatively different payoff relationships for Disease 1 and Disease 2. The heat maps in (A)–(D) correspond to Disease 1 and the heat maps in (E)–(H) correspond to Disease 2. In (A) and (E) the payoff of being susceptible and recovered are equal, which means that agents recover completely from the disease after infection. In (B) and (F), the recovered payoff is lower than the susceptible payoff, but still greater than the prophylactic payoff meaning that being recovered is preferable to than being in the prophylactic state. In (C) and (G), the payoff for the prophylactic and recovered states are exactly equal. In (D) and (G), the disease is permanently debilitating such that the payoff of the recovered state is less than the prophylactic state. The heat maps of behavioral change assume the following payoffs for *u*_S_, *u*_P_, *u*_I_, *u*_R_. (A):{1, 0.95, 0.1, 1}, (B):{1, 0.95, 0.1, 0.97}, (C):{1, 0.95, 0.1, 0.95}, (D):{1, 0.95, 0.1, 0.9}, (E):{1, 0.95, 0.6, 1}, (F):{1, 0.95, 0.6, 0.97}, (G):{1, 0.95, 0.1, 0.95}, and (H):{1, 0.95, 0.1, 0.9}. The % Protection corresponds to the percentage of protection obtained for adopting prophylactic behavior, (1 − *ρ*) × 100.

Returning our focus to Fig. 2D, region *B* corresponds to the situation in which agents will adopt non-prophylactic or prophylactic behavior depending on the prevalence of the disease (Fig. 2B). If the disease prevalence is smaller than the switching point, the agent opts for the susceptible behavior; otherwise it adopts the prophylactic behavior. Region *C* corresponds to the situation in which two switching points exist instead of a single one (Fig. 2C). When the proportion of infectious agents is between these switching points, agents adopt prophylactic behavior, while values outside this range drives agents to adopt non-prophylactic behavior. This situation is of particular interest because it shows that the adoption of prophylactic behavior is not always monotonically associated with the prevalence of the disease.

The utility calculations that agents use to decide whether to adopt a behavior are complex (see Eqs. (9) and (10)); an exhaustive exploration of the parameter space is not undertaken here. We instead investigate several paradigm cases related to the payoff ordering. We assume that the payoff for the infectious state (*u*_I_) relies upon biological parameters of the disease and always corresponds to the lowest payoff, thus we need only consider the relationship between the other three payoffs. In particular, we are interested in looking at situations where the recovery payoff ranges from complete recovery (case 1) to less than the prophylactic state (case 4).

Case 1: *u*_S_ = *u*_R_ > *u*_P_ > *u*_I_,
Case 2: *u*_S_ > *u*_R_ > *u*_P_ > *u*_I_,
Case 3: *u*_S_ > *u*_R_ = *u*_P_ > *u*_I_, and
Case 4: *u*_S_ > *u*_P_ > *u*_R_ > *u*_I_.

Because our model consists of a constant population of *N* agents (i.e., no mortality), cases in which *u*_S_ > *u*_R_ represent situations where an individual suffers chronic harm from the disease.

Figure 4 displays the switching point heat maps for these different ordering cases of Disease 1 (Figs. 4A–4D) and Disease 2 (Figs. 4E–4H). The most dramatic difference between the two diseases is that changing the payoff for being recovered has a large effect on the agents’ behavioral change in the cases of Disease 2, but little effect in the case of Disease 1. The reason for this has to do with the biological parameters of the model, in particular, the disease recovery time for Disease 1 is large (Recovery Time = 65), but small for Disease 2 (Recovery Time = 8). Consequently, an agent expects to spend more time in the recovered state when considering Disease 2 than Disease 1. When weighting these expected times with different payoffs for calculating the utilities, there will be less variation in Disease 1 compared with Disease 2.

The effects of progressively reducing the recovered payoff are more evident for Disease 2. Reducing the recovered payoff means that lower levels of prevalence will be sufficient for agents to change their behavior. In the case of equal value for recovered and susceptible payoffs, agents consider changing behavior only in narrow parameter range of protection efficacy and planning horizon values (Fig. 4E). Progressively reducing the recovered payoff, i.e., moving from case 1 (Fig. 4E) to case 4 (Fig. 4H), the range of parameter values that induce agents to change their behavior expands (i.e., there are large areas of the parameter space in which the agents would consider changing behavior) and the disease prevalence necessary for such change to occur decreases (i.e., gradual change of the color towards blue).

In addition to this numerical analysis, we have also obtained analytical results for case 2 (payoff ordering *u*_S_ > *u*_R_ > *u*_P_ > *u*_I_) that are available in Sec. S4 Supplemental Information 1.

### Epidemic dynamics

We turn now to understand how the above conditions for behavioral change may influence epidemic dynamics. Here we are particularly interested in analyzing the effects of the planning horizon H and the decision frequency *δ* on the dynamics of Disease 1 and Disease 2. Because we assume that the interactions among the population are well-mixed, we execute the simulations using the ODE model for a population of 100, 000 agents (initially 99, 900 agents in the susceptible state and 100 in the infectious state) with a decision frequency of *δ* = 0.01.

**Figure 5:**
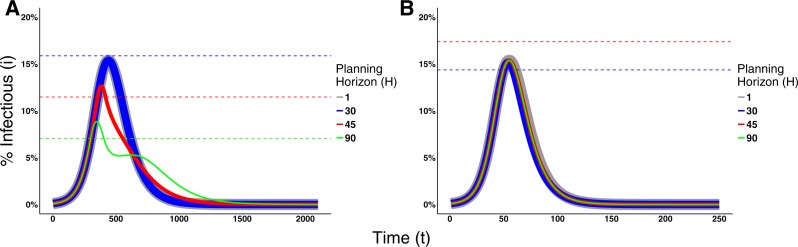
Effects of the Planning Horizon on the epidemic dynamics. The solid lines represent the proportion of infectious agents in the population over time. The line thickness is not meaningful; rather, it is used to facilitate the visualization due to the fact that the dynamics overlap each other. The dashed lines represent the switching point associated to the planning horizon reported with the same color in the legend. Missing dashed lines indicate that no switching point exists for that planning horizon. (A) Epidemic dynamics for Disease 1 with payoffs {1, 0.95, 0.1, 0.95} and *ρ* = 0.1. (B) Epidemic dynamics for Disease 2 with payoffs {1, 0.95, 0.6, 1} and *ρ* = 0.01. Both dynamics consider decision frequency *δ* = 0.01 for planning horizons 1, 30, 45, and 90.

Figure 5 shows the effects of different planning horizons on the epidemic dynamics for both Disease 1 (Fig. 5A) and Disease 2 (Fig. 5B). For short planning horizons (i.e., H = 1), agents do not ever consider changing behavior in either disease. This corresponds to the situation in Region *A* in Fig. 2A in which being prophylactic is never worth the cost, hence the epidemic dynamics are not affected. Similarly, in the cases of H = 30 for Disease 1 and H = 45 for Disease 2, we notice that neither of the two epidemic dynamics change. The dynamics are not affected because the disease prevalence does not reach the switching point (the switching points are indicated by the dashed lines in Fig. 5).

In the cases of H = 45 and 90 for Disease 1 and H = 30 for Disease 2, however, agents change behavior and thereby affect epidemic dynamics. For Disease 1, the effect is characterized by the decrease on the epidemic peak size and a prolonged duration of the epidemic. Although the dynamics of Disease 2 are also affected, the effect is small because a lower portion of the population crosses the switching point.

In other cases, increasing the planning horizon further may cause agents to never contemplate a change in their behavior. This occurs, for example, when H = 90 for Disease 2. This means that agents willingly assume the risk of getting infected and then recover, which is intuitive given the short recovery time and mild severity of the disease.

**Figure 6:**
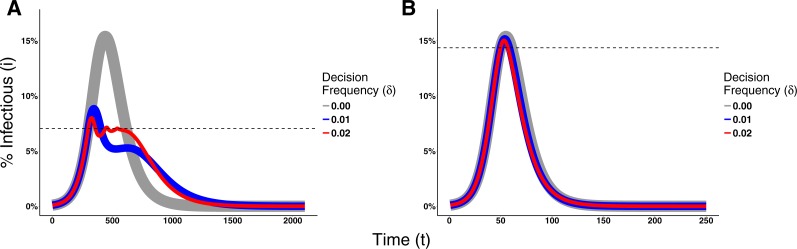
Effects of the Decision Frequency on the epidemic dynamics. The solid lines represent the proportion of infectious agents in the population over time. The line thickness is not meaningful; rather, it is used to facilitate the visualization due to the fact that the dynamics overlap each other. The dashed lines represent the switching point of H = 90 for Disease 1 and H = 30 for Disease 2. (A) Epidemic dynamics for Disease 1 with payoffs {1, 0.95, 0.1, 0.95} and *ρ* = 0.1. (B) Epidemic dynamics for Disease 2 with payoffs {1, 0.95, 0.6, 1} and *ρ* = 0.01.

To assess the effect of the frequency that agents make behavioral decisions on the epidemic dynamics, we fix the value of the planning horizon for Disease 1 (H = 90) and Disease 2 (H = 30), and vary the decision rate. Figure 6 shows the effects of different decision frequencies on the epidemic dynamics. This figure illustrates how increasing the decision frequency reduces the epidemic peak size while prolonging the epidemic. It additionally may generate multiple waves of infection for Disease 1. These waves are generated because raising the decision frequency means individuals react faster to an increase in prevalence and adopt the prophylactic behavior. This bends the trajectory of disease incidence downward, but the reduction in prevalence causes the pendulum to swing back and individuals return back to their non-prophylactic behavior, thus creating an environment for the resurgence of the epidemic.

### Risk perception

Empirical evidence shows that humans change behavior and adopt costly preventative measures, even if disease prevalence is low. This is especially true for harmful diseases with severe consequences to those being infected, such as Ebola or the Severe Acute Respiratory Syndrome (SARS). For example, despite the low level of recorded cases during the 2003 SARS outbreak in China (approximately 5,327 cases), people in the city of Guangzhou avoided going outside or wore masks when outside (World Health Organization, 2003; Tai & Sun, 2007). Combined with the severity of the disease, other factors like misinformation or excess media coverage may distort the perception of disease prevalence (i.e., risk perception), making individuals respond unexpectedly to an epidemic.

Several models incorporate specific mechanisms regulating the diffusion of information about the disease to understand the above factors and how they contribute to the distortion of risk perception (Epstein et al., 2008; Funk et al., 2009; Poletti, Ajelli & Merler, 2012). Here our focus is slightly different. We are interested in understanding the effects that such distorted perception has on the epidemic dynamics. Thus in our model, we have incorporated a *distortion factor κ* that alters the agents’ perception about disease prevalence used in calculating their utilities. For *κ* = 1, agents have the true perception of the disease prevalence; for *κ* > 1, the perceived disease prevalence is inflated and *κ* reflects an increase in the risk perception of being infected; for *κ* < 1, the perceived disease prevalence is reduced below its true value.

**Figure 7:**
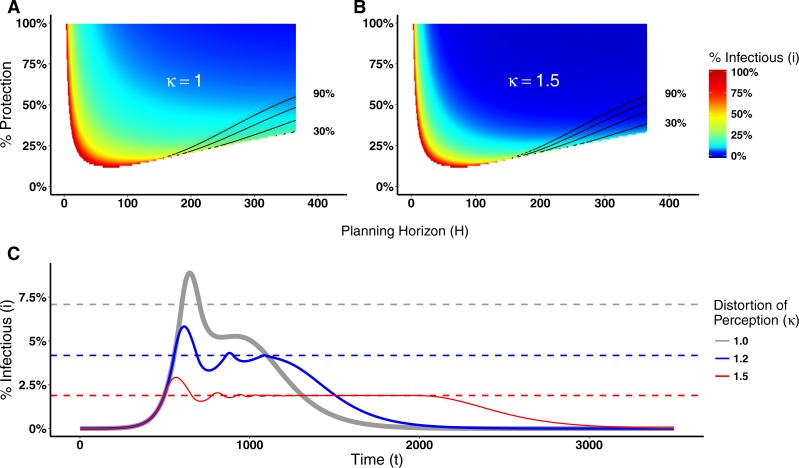
Heat maps of switching points and epidemic dynamics for Disease 1. (A) and (B) show the proportion of infectious agents above which the prophylactic behavior is more advantageous than the non-prophylactic behavior given the percentage of protection obtained for adopting the prophylactic behavior (1 − *ρ*) × 100 and the planning horizon H. See Fig. 2 for more details on interpreting switching point heat maps. (A) No perception distortion: *κ* = 1; (B) A distortion factor *κ* = 1.5, which reduces the proportion of infectious agents above which the prophylactic behavior is more advantageous. (C) Epidemic dynamics for different distortion factors show how increasing *κ* reduces the epidemic peak size, prolongs the epidemic and generates secondary waves of infection.

Distorting the perception of a disease prevalence can lead to changes in the decision making process, and consequently on epidemic dynamics, as illustrated in Fig. 7 for Disease 1 (see Fig. S2 for Disease 2). Figure 7A shows the switching point assuming agents know the real disease prevalence (*κ* = 1). By distorting the perceived disease prevalence (*κ* = 1.5), the real proportion of infectious agents necessary for agents to engage in prophylactic behavior is reduced as shown in Fig. 7B. Hence, the distortion on disease prevalence makes agents engage in prophylactic behavior even when the chance of being infected is low. This affects the epidemic dynamics by reducing the epidemic peak size but prolonging the epidemic and generating multiple waves of infection as shown in Fig. 7C.

## Discussion

Individuals acting in their own self-interest make behavioral decisions to reduce their likelihood of getting infected in response to an epidemic. We explore a decision making process that integrates the prophylaxis efficacy and the current disease prevalence with individuals’ payoffs and planning horizon to understand the conditions in which individuals adopt prophylactic behavior.

Our results show that the adoption of prophylactic behavior is sensitive to a planning horizon. Individuals with a short planning horizon (i.e., “live for the moment”) do not engage in prophylactic behavior because of its adoption costs. Individuals with a long planning horizon also fail to adopt prophylactic behavior, but for different reasons. They prefer to “get it over with” and enjoy the benefits of being recovered. In both these situations, the epidemic dynamics remain unchanged because the individuals do not have an incentive to engage in prophylactic behavior even when the disease prevalence is high. For intermediate planning horizons, however, individuals adopt prophylactic behavior depending on the disease parameters and the prophylaxis efficacy. The effects on disease dynamics include a reduction in the epidemic peak size, but a prolonged epidemic.

The time scale of a planning horizon (i.e., what constitutes short, intermediate, and long), however, depends on the disease parameters. While the time scale for Disease 2 is on the order of days, for Disease 1 the time scale is on the order of months to years.

These results are consistent with the findings of Fenichel et al. (2011), who also concluded that behavioral change is sensitive to a planning horizon. The SPIR and Fenichel et al. models generate similar results, but differ in several aspects. In the latter, susceptible agents optimize their contact rate by balancing the expected incremental benefits and costs of additional contacts. Moreover, the agents take into consideration only the payoffs of being susceptible and recovered when optimizing the contact rates. In the SPIR model, however, agents maintain a constant contact rate, yet adopt prophylactic behavior that reduces the chance of getting infected. When agents are deciding to engage in prophylactic behavior, they take into account the payoff of all possible epidemiological states. The fact that we reach the same conclusion using different models further supports the claim that the planning horizon is a relevant decision making factor in understanding epidemic dynamics.

Although associated with the prevalence of disease, the adoption of prophylactic behavior is not always monotonically associated with it. Its adoption depends on the behavioral decision parameters. For severe diseases with long recovery times, e.g., Disease 1, the option of prophylactic behavior is less sensitive to changes in the payoffs (Figs. 4A–4D) compared to less severe diseases with shorter recovery times, e.g., Disease 2 (Figs. 4E–4H). This implies that understanding the payoffs related to each disease is critical to proposing effective public policies, especially because there is not a “one-size-fit-all” solution.

Another aspect to highlight is that the beneficial adoption of prophylactic behavior can be achieved through two different public policies: changing the risk perception or introducing incentives that reduce the difference between the susceptible and prophylactic payoffs (i.e., reduce the cost of adopting prophylactic behavior). One problem with increasing the risk perception is that if it is overdone, it leads, in some situations, to the opposite result to the one that is desired. Because individuals perceive their chance of getting the disease as highly probable, they prefer to “get it over with” and enjoy the benefits of being recovered. In contrast, the more the prophylaxis is incentivized the better the results, e.g., reduction of epidemic peak size.

Similar to our SPIR model, Perra et al. (2011) and Del Valle, Mniszewski & Hyman (2013) also proposed an extension to the SIR model and included a new compartment that reduces the transmission rate between the susceptible and infectious states. A clear distinction between these models and the SPIR model is that their agents do not take into account the costs associated with moving between the susceptible compartment and this new compartment. While in Perra et al. (2011) agents make the decision to move between compartments based on the disease prevalence, in Del Valle, Mniszewski & Hyman (2013) constant transfer rates are defined to handle the transition.

In addition to these differences, an advantage of the SPIR model with respect to all other models that implement some behavioral change is the distinction between the disease dynamics model and the behavioral decision model. This distinction renders the SPIR model flexible by making it easier to, for example, implement other decision making processes. Consequently, this modular and flexible model architecture facilitates the execution of comparative studies with different behavioral decision models coupled to a common epidemiological model, which we plan to perform as future work. For example, an interesting avenue of investigation is to relax our assumptions about rationality. There are a number of psychosocial theories that have been developed to predict, explain, and change health behaviors, such as the Health Belief Model (Becker, 1974) and the Social Cognition Theory (Bandura, 1986). The effects of these kinds of decision-making processes on epidemic dynamics have not been fully explored.

## Supplemental Information

10.7717/peerj.2678/supp-1 Supplemental Information 1 **** Raw data, scripts, figures, and abm source-code files

## Additional Information and Declarations

### Data Availability

The following information was supplied regarding data availability: The raw data has been supplied as Supplemental Information 1.
