# Supplementary material for: Planning horizon affects prophylactic decision-making and epidemic dynamics
Source: PeerJ. 2016 Nov 8;4:e2678. doi: 10.7717/peerj.2678 (PMC5103819; doi:10.7717/peerj.2678)
Supplement: Supplemental Information 1 [file peerj-04-2678-s001.zip › spir-paper-si/figures/figure4.pdf]

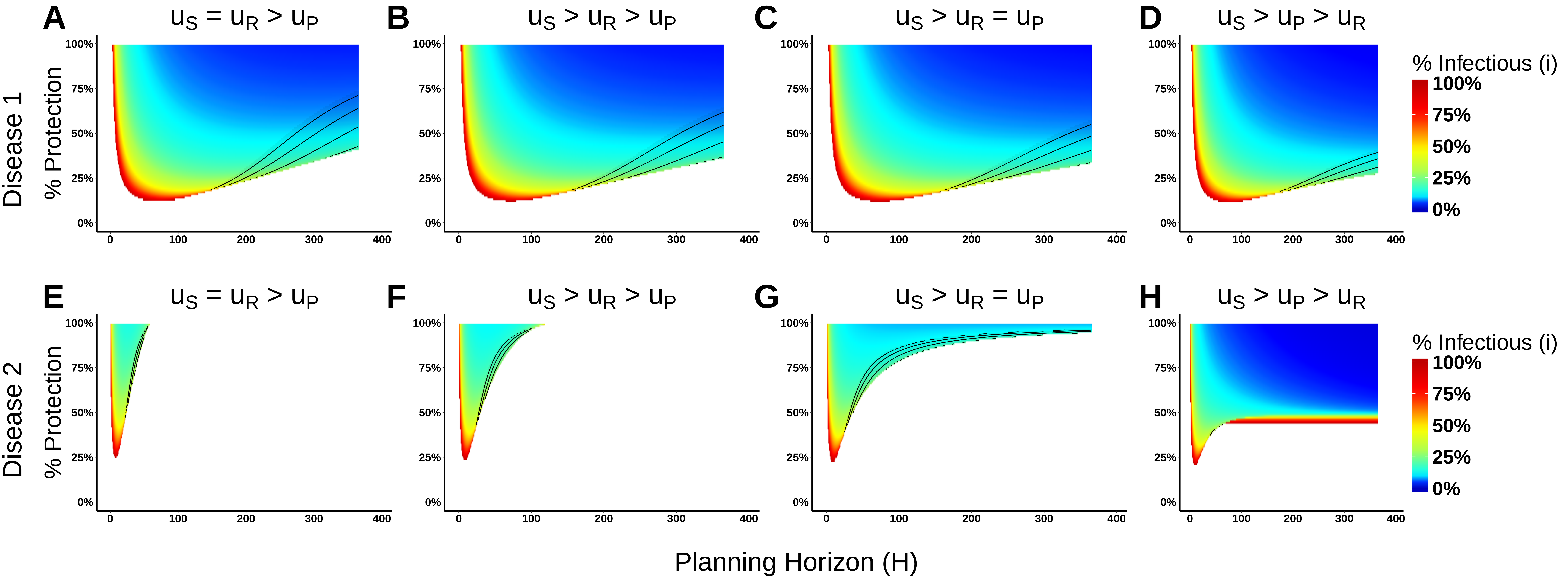

A
$u_S = u_R > u_P$
B
$u_S > u_R > u_P$
C
$u_S > u_R = u_P$
D
$u_S > u_P > u_R$
E
$u_S = u_R > u_P$
F
$u_S > u_R > u_P$
G
$u_S > u_R = u_P$
H
$u_S > u_P > u_R$
Disease 1
Disease 2
% Protection
100%
75%
50%
25%
0%
0
100
200
300
400
% Infectious (i)
100%
75%
50%
25%
0%
Planning Horizon (H)
