# Supplementary material for: Planning horizon affects prophylactic decision-making and epidemic dynamics
Source: PeerJ. 2016 Nov 8;4:e2678. doi: 10.7717/peerj.2678 (PMC5103819; doi:10.7717/peerj.2678)
Supplement: Supplemental Information 1 [file peerj-04-2678-s001.zip › spir-paper-si/supplemental_information.pdf]

Supplemental Information to
Planning horizon affects prophylactic decision-making and epidemic dynamics

Luis G. Nardin, Craig R. Miller, Benjamin J. Ridenhour, Stephen M. Krone, Paul Joyce, and Bert O. Baumgaertner

Content

This Supplemental Information document contains information about:

Section S1: Content of folders

Section S2: Supporting figures

Section S3: $\mathrm{E}\left[T_{\mathrm{I}|\mathrm{D_X}}\right]$ derivation

Section S4: Analytical behavioral decision analysis

Section S5: Brief description and instruction of use of the R scripts

Section S6: Pre-requisites, configuration, and usage of the agent-based model source-code

Section S7: Comparison between the ABM and ODE results

S1 Content of Folders

The Supplemental Information file has several directories containing all the raw data and code used during the development of the paper. Table S1 describes briefly the content of each of the folders.

Table S1. Description of the folders.

**Folder name**	**Description**
scripts	R scripts used to generate data and figures (see Sec. S5).
data	Raw data used to generate the figures and perform the analysis.
figures	Figure files generated in PDF format.
code	Agent-based model source-code (see Sec. S6).
config	ABM configuration file used to generate the results in Sec. S7.

13 S2 Supporting Figures

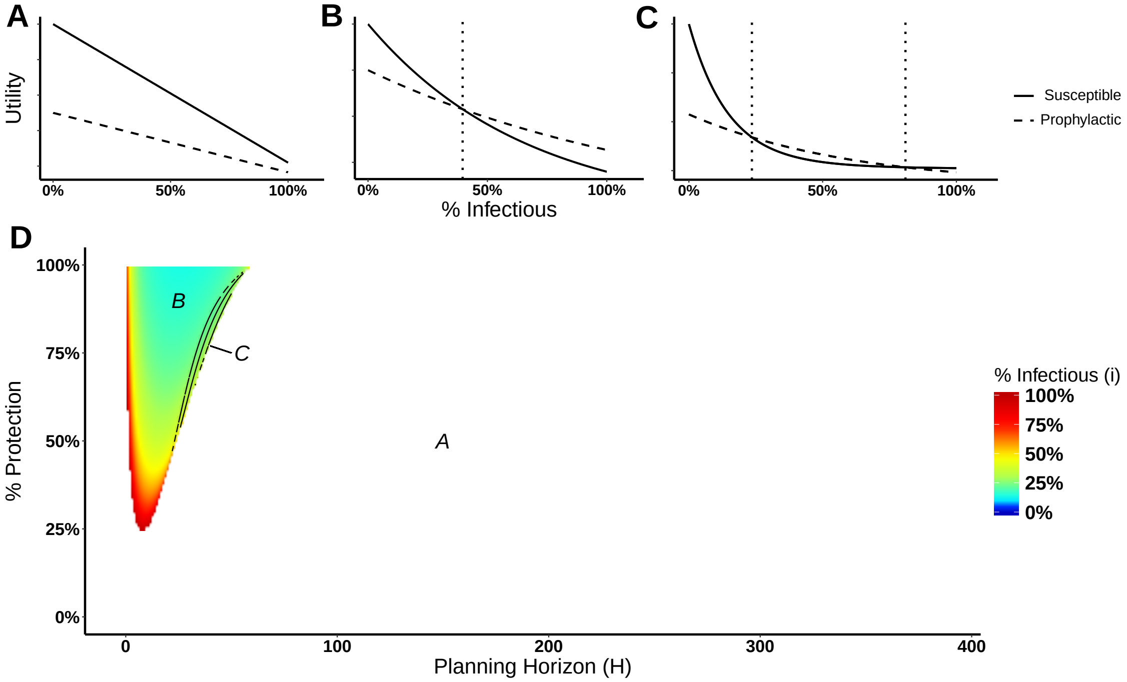

Figure S1. Heat map of switching points for Disease 2.
(A) Situation in which non-prophylactic behavior is always more advantageous than prophylactic behavior regardless of the proportion of infectious agents. (B) Situation in which above a certain proportion of infectious agents (i.e. value indicated by the vertical dotted line), the prophylactic behavior is more advantageous than non-prophylactic behavior. (C) Situation in which prophylactic behavior is more advantageous whenever the proportion of infectious agents is within a range of values represented by the two vertical dotted lines and less advantageous otherwise. (D) Proportion of infectious agents above which prophylactic behavior is more advantageous than non-prophylactic behavior given the percentage of protection (% Protection) obtained for adopting prophylactic behavior $(1-\rho)\times 100$ (y-axis) and the planning horizon H (x-axis). The three regions in (D) represent the situations shown in panels (A), (B), and (C). In region *A*, agents never adopt prophylactic behavior. In region *B*, agents adopt prophylactic behavior above the reported proportion of infectious agents. In region *C*, agents adopt prophylactic behavior only if the proportion of infected agents are between the proportion of infectious agents represented by the color gradient and the proportion represented by the contour lines.

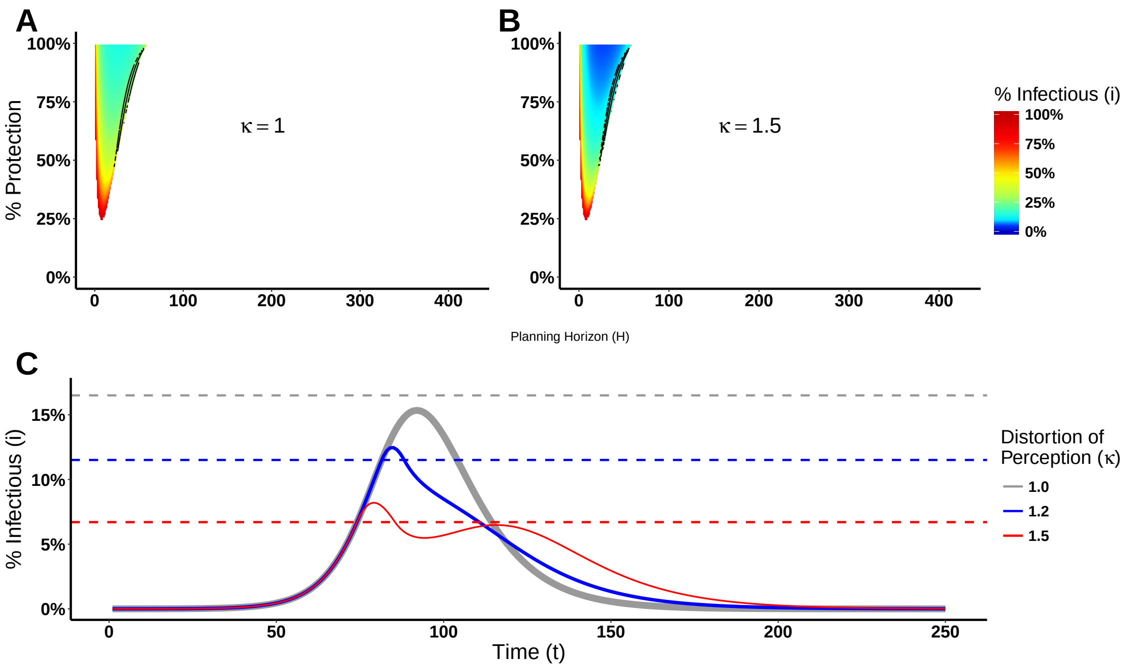

Figure S2. **Heat maps of switching points and epidemic dynamics for Disease 2.** (A) and (B) show the proportion of infectious agents above which the prophylactic behavior is more advantageous than the non-prophylactic behavior given the percentage of protection obtained for adopting the prophylactic behavior $1-\rho) \times 100$ (y-axis) and the planning horizon H (x-axis). For more information about interpreting switching point heat maps see Fig. S1. (A) No perception distortion: $\kappa = 1$; while (B) Distortion factor $\kappa = 1.5$, which reduces the proportion of infectious agents above which the prophylactic behavior is more advantageous. (C) Epidemic dynamics for different distortion factors shows how increasing κ reduces the epidemic peak size and prolongs the epidemic.

S3 $\mathrm{E}\left[T_{\mathbf{I}|\mathbf{D_X}}\right]$ derivation

Given that the expected time spent in state $\mathrm{X} \in \{S, P\}$ can be expressed as

$$\mathrm{E}\left[T_{\mathrm{X}|\mathrm{D_X}}\right] = \left(\frac{1}{f_\mathrm{X}} - 1\right)\left(1 - (1 - f_\mathrm{X})^{\mathrm{H}}\right), \tag{1}$$

we derive the expected time spent in I by conditioning on $T_{\mathrm{X}|\mathrm{D_X}}$

$$\mathrm{E}\left[T_{\mathrm{I}|\mathrm{D_X}}\right] = \mathrm{E}\left[\mathrm{E}\left[T_{\mathrm{I}|\mathrm{D_X}} | T_{\mathrm{X}|\mathrm{D_X}}\right]\right]. \tag{2}$$

The conditional expectation is of the same form as Eq. (1), but with f_X replaced by g and H replaced by the remaining time $\mathrm{H} - T_{\mathrm{X}|\mathrm{D_X}}$. Thus,

$$\begin{aligned}
\mathrm{E}\left[T_{\mathrm{I}|\mathrm{D_X}}\right] &= \mathrm{E}\left[\left(\frac{1}{g} - 1\right)\left(1 - (1-g)^{\mathrm{H} - T_{\mathrm{X}|\mathrm{D_X}}}\right)\right] \\
&= \left(\frac{1}{g} - 1\right)\mathrm{E}\left[1 - (1-g)^{\mathrm{H} - T_{\mathrm{X}|\mathrm{D_X}}}\right] \\
&= \left(\frac{1}{g} - 1\right)\left(1 - \left[\sum_{T_{\mathrm{X}|\mathrm{D_X}}=0}^{\mathrm{H}-1} (1-g)^{\mathrm{H} - T_{\mathrm{X}|\mathrm{D_X}}} f_\mathrm{X} (1-f_\mathrm{X})^{T_{\mathrm{X}|\mathrm{D_X}}} + (1-f_\mathrm{X})^{\mathrm{H}}\right]\right) \\
&= \left(\frac{1}{g} - 1\right)\left(1 - (1-f_\mathrm{X})^{\mathrm{H}} - \frac{f_\mathrm{X}(1-g)^{\mathrm{H}+1}}{f_\mathrm{X} - g}\left[1 - \left(\frac{1-f_\mathrm{X}}{1-g}\right)^{\mathrm{H}}\right]\right) \\
&= \left(\frac{1}{g} - 1\right)\left(1 - (1-f_\mathrm{X})^{\mathrm{H}} - \frac{f_\mathrm{X}(1-g)^{\mathrm{H}+1}}{f_\mathrm{X} - g} + \frac{(1-f_\mathrm{X})^{\mathrm{H}}}{(1-g)^{\mathrm{H}}}\frac{f_\mathrm{X}(1-g)^{\mathrm{H}+1}}{f_\mathrm{X} - g}\right) \\
&= \left(\frac{1}{g} - 1\right)\frac{1}{f_\mathrm{X} - g}\left[\left(1 - (1-f_\mathrm{X})^{\mathrm{H}}\right)(f_\mathrm{X} - g) - f_\mathrm{X}(1-g)^{\mathrm{H}+1} + (1-f_\mathrm{X})^{\mathrm{H}} f_\mathrm{X}(1-g)\right] \\
&= \left(\frac{1}{g} - 1\right)\frac{1}{f_\mathrm{X} - g}\left(f_\mathrm{X} - g + (1-f_\mathrm{X})^{\mathrm{H}+1} g - f_\mathrm{X}(1-g)^{\mathrm{H}+1}\right) \\
&= \left(\frac{1}{g} - 1\right)\frac{f_\mathrm{X}}{f_\mathrm{X} - g}\left(1 - \frac{g}{f_\mathrm{X}}\left(1 - (1-f_\mathrm{X})^{\mathrm{H}+1}\right) - (1-g)^{\mathrm{H}+1}\right) \\
&= \left(\frac{1}{g} - 1\right)\frac{f_\mathrm{X}}{f_\mathrm{X} - g}\left(1 - (1-g)^{\mathrm{H}+1} - \frac{g}{f_\mathrm{X}}\left(1 - f_\mathrm{X}(1-f_\mathrm{X})^{\mathrm{H}+1}\right) - g\right)
\end{aligned}$$

$$= \frac{f_X(1-g)}{f_X - g}\left[\left(\frac{1-g}{g} - \frac{(1-g)^{H+1}}{g}\right) - \left(\frac{1-f_X}{f_X} - \frac{(1-f_X)^{H+1}}{f_X}\right)\right]$$

Since $\frac{f_X(1-g)}{f_X-g} = \left(\frac{1}{g}-1\right)\left(\frac{1}{\left(\frac{1}{g}-1\right)-\left(\frac{1}{f_X}-1\right)}\right)$, we conclude that

$$\mathrm{E}\left[T_{I|D_X}\right] = \left(\frac{1}{g}-1\right)\left(\frac{\left(\frac{1}{g}-1\right)\left(1-(1-g)^{H}\right) - \left(\frac{1}{f_X}-1\right)\left(1-(1-f_X)^{H}\right)}{\left(\frac{1}{g}-1\right)-\left(\frac{1}{f_X}-1\right)}\right). \tag{3}$$

S4 Analytical behavioral decision analysis

In addition to the numerical analysis presented in Behavioral Decision Analysis, we have also obtained analytical results for case 2 (payoff ordering $u_S > u_R > u_P > u_I$) to identify the general conditions necessary for the existence of one or more switching points. Mathematically, switching points occur where the utility functions for S and P are equal (Eqs. 9 and 10). Replacing the expected time notation $\mathrm{E}\left[T_{Y|D_X}\right]$ in the utility U_S and U_P by the more concise $\overline{T}_{Y|X}$, where $X \in \{S,P\}$ and $Y \in \{S,P,I,R\}$, we have

$$u_S\overline{T}_{S|S} + u_I\overline{T}_{I|S} + u_R\overline{T}_{R|S} = u_P\overline{T}_{P|P} + u_I\overline{T}_{I|P} + u_R\overline{T}_{R|P}.$$

Given that $\overline{T}_{R|X} = H - \overline{T}_{X|X} - \overline{T}_{I|X}$,

$$\begin{aligned}
u_S\overline{T}_{S|S} + u_I\overline{T}_{I|S} + u_R\left(H - \overline{T}_{S|S} - \overline{T}_{I|S}\right) &= u_P\overline{T}_{P|P} + u_I\overline{T}_{I|P} + u_R\left(H - \overline{T}_{P|P} - \overline{T}_{I|P}\right)\\
(u_S - u_R)\overline{T}_{S|S} + (u_I - u_R)\overline{T}_{I|S} &= (u_P - u_R)\overline{T}_{P|P} + (u_I - u_R)\overline{T}_{I|P}\\
\left(\frac{u_S - u_R}{u_I - u_R}\right)\overline{T}_{S|S} + \overline{T}_{I|S} &= \left(\frac{u_P - u_R}{u_I - u_R}\right)\overline{T}_{P|P} + \overline{T}_{I|P}.
\end{aligned}$$

Let $K_1 = \frac{u_S - u_R}{u_I - u_R}$, which weights the benefits of S and I, and $K_2 = \frac{u_P - u_R}{u_S - u_R}$, which weights the benefit of S and P. Then,

$$\begin{aligned}
K_1\overline{T}_{S|S} + \overline{T}_{I|S} &= K_1K_2\overline{T}_{P|P} + \overline{T}_{I|P}\\
\overline{T}_{I|S} - \overline{T}_{I|P} &= K_1\left(K_2\overline{T}_{P|P} - \overline{T}_{S|S}\right)\\
K_1^{-1}\left(\overline{T}_{I|S} - \overline{T}_{I|P}\right) &= K_2\overline{T}_{P|P} - \overline{T}_{S|S}.
\end{aligned}$$

Because the payoff ordering $u_S > u_R > u_P > u_I$ and noting that $\overline{T}_{I|S} \geq \overline{T}_{I|P}$ and $K_1^{-1} = \frac{u_I - u_R}{u_S - u_R}$, we have that $K_1^{-1}\left(\overline{T}_{I|S} - \overline{T}_{I|P}\right) \leq 0$ and $0 < K_2 < 1$. From these we analyze some general cases.

First, we analyze the case in which $K_2\overline{T}_{P|P} - \overline{T}_{S|S} \geq 0$, then $K_2\overline{T}_{P|P} \geq \overline{T}_{S|S}$. Because $K_1^{-1}\left(\overline{T}_{I|S} - \overline{T}_{I|P}\right) \leq 0$, there never is a switching point and agents will strictly opt for prophylaxis (i.e. state P).

A more interesting case is when the planning horizon H is long enough to produce the condition where $\overline{T}_{\mathrm{I|S}} \approx \overline{T}_{\mathrm{I|P}} \approx \frac{1}{g} - 1$ implying that

$$\begin{aligned}
\mathrm{K}_2\overline{T}_{\mathrm{P|P}} - \overline{T}_{\mathrm{S|S}} &= \mathrm{K}_1^{-1}\left(\overline{T}_{\mathrm{I|S}} - \overline{T}_{\mathrm{I|P}}\right) \\
\mathrm{K}_2\overline{T}_{\mathrm{P|P}} - \overline{T}_{\mathrm{S|S}} &= 0 \\
\mathrm{K}_2\left(\frac{1}{\rho f_{\mathrm{X}}} - 1\right) - \left(\frac{1}{f_{\mathrm{X}}} - 1\right) &= 0 \\
\mathrm{K}_2\frac{1}{\rho f_{\mathrm{X}}} &= \frac{1}{f_{\mathrm{X}}} \\
\mathrm{K}_2 &= \rho.
\end{aligned}$$

This case always produces a switching point and occurs when $1-(1-f_{\mathrm{X}})^{\mathrm{H}} \approx 1 \implies (1-f_{\mathrm{X}})^{\mathrm{H}} \approx 0$.

Note that if f_{X} is large (i.e. $\frac{1}{f_{\mathrm{X}}} \approx 0$) or $f_{\mathrm{X}} \approx \rho f_{\mathrm{X}}$, then a switching point hypothetically exists because it produces a condition where $\overline{T}_{\mathrm{S|S}} = \overline{T}_{\mathrm{P|P}}$. However, in these cases $0 = \mathrm{K}_2\overline{T}_{\mathrm{P|P}} - \overline{T}_{\mathrm{S|S}} = \mathrm{K}_2\overline{T}_{\mathrm{S|S}} - \overline{T}_{\mathrm{S|S}} \implies \mathrm{K}_2 = 1$ and because $0 < \mathrm{K}_2 < 1$, a switching point never exists.

The last case occurs when H assumes an intermediate planning horizon. When $\mathrm{K}_2\overline{T}_{\mathrm{P|P}} - \overline{T}_{\mathrm{S|S}} < 0$, we can conclude that $\mathrm{K}_2 < \frac{\overline{T}_{\mathrm{S|S}}}{\overline{T}_{\mathrm{P|P}}}$ for the switching point to exist in the non-limiting case (i.e. when $\mathrm{K}_1^{-1}\left(\overline{T}_{\mathrm{I|S}} - \overline{T}_{\mathrm{I|P}}\right) \not\approx 0$). For example, if the payoff relative to recovery of P versus S is 0.5, then agents must expect less than twice the time in P to consider switching. Assuming that $\mathrm{K}_2\overline{T}_{\mathrm{P|P}} - \overline{T}_{\mathrm{S|P}} < 0$, we must then further meet the condition $\mathrm{K}_1^{-1}\left(\overline{T}_{\mathrm{I|S}} - \overline{T}_{\mathrm{I|P}}\right) = \mathrm{K}_2\overline{T}_{\mathrm{P|P}} - \overline{T}_{\mathrm{S|S}}$ to get a switching point; this occurs when

$$\mathrm{K}_2 = \frac{\mathrm{K}_1^{-1}\left(\overline{T}_{\mathrm{I|S}} - \overline{T}_{\mathrm{I|P}}\right) - \overline{T}_{\mathrm{S|S}}}{\overline{T}_{\mathrm{P|P}}} = \frac{\mathrm{K}_1^{-1}\left(\overline{T}_{\mathrm{I|S}} - \overline{T}_{\mathrm{I|P}}\right)}{\overline{T}_{\mathrm{P|P}}} - \frac{\overline{T}_{\mathrm{S|S}}}{\overline{T}_{\mathrm{P|P}}}.$$

S5 Scripts

Table S2 describes the R Statistics scripts used to generate data and figures shown in the paper.

Table S2. R scripts function description.

Script Filename	Description
calcExpectedTime.R	Implements the function that calculates the expected times $\mathrm{E}\left[T_{\mathrm{X}\mid\mathrm{D_Y}}\right]$, where $\mathrm{X} \in \{\mathrm{S},\mathrm{P},\mathrm{I},\mathrm{R}\}$ and $\mathrm{Y} \in \{\mathrm{S},\mathrm{P}\}$ given a set of conditions.<br>**Function:** *calc_expectedTime(h, i, bs, rho, g, l, k, payoffs)*
calcSwitch.R	Implements the function that calculates the switching points given a set of conditions.<br>**Function:** *calc_iswitch(h, bs, rho, g, l, k, payoffs)*
calcUtilities.R	Implements the function that calculates the utilities given a set of conditions.<br>**Function:** *calc_utilities(h, bs, rho, g, l, k, payoffs)*
SPIRmodel.R	Implements the function SPIR ODE model.<br>**Function:** *SPIRmodel(Time, State, Pars)*
disease1-data.R	Script that generates the data used to create the figures of Disease 1.
disease2-data.R	Script that generates the data used to create the figures of Disease 2.
figure2.R	Script that recreates figure of the heat map of switch points for Disease 1.
figure3.R	Script that recreates the figure of expected proportion of planning horizon spent in each state.
figure4.R	Script that recreates the heat map of switching points for payoffs ordering cases of Disease 1 and Disease 2.
figure5.R	Script that recreates figure effects of the planning horizon on the epidemics dynamics.
figure6.R	Script that recreates figure effects of the decision frequency on the epidemics dynamics.
figure7.R	Script that recreates figure heat maps of switching points and epidemics dynamics for Disease 1 with distortion different than factor.

These scripts require `R v.3.3.1` with the following libraries: `colorRamps v.2.3`, `data.table v.1.9.6`, `deSolve v.1.13`, `doParallel v.1.0.10`, `ggplot2 v.2.1`, `grid v.3.3.1`, and `gridExtra v.2.2.1`, and `parallel v.3.3.1`.

To execute the scripts from Linux terminal:

- Navigate to the `scripts` directory
- Execute: Rscript <Script Filename> --no-save, where <Script Filename> is the script filename shown in column **Script Filename** of Table S2.

S6 Agent-Based Model User's Guide

S6.1 Prerequisites

The SPIR agent-based model version is written in `Python 3`, thus it can be run on any Python-supported operating system. In addition to `Python 3.0+`, this implementation uses a set of existing standard libraries that needs to be installed prior to its execution:

- `argparse`
- `matplotlib`
- `os.path`
- `sys`
- `time`

S6.2 Download from GitHub

The directory *code* in the Supplemental Information material contains the SPIR agent-based model source-code. However, we release the most up-to-date version of the SPIR ABM model in the GitHub (`http://www.github.com`). To download the SPIR source-code

- Open a Linux terminal
- Navigate to the directory where you want to download it
- Type
  `git clone git@github.com:bertybaums/SPIR.git`
  or
  `git clone https://github.com/bertybaums/SPIR.git`

S6.3 Usage

Syntax: `Main.py [-h] [-v] [-o] [-g] {configFile, params} ...`
where,

`-h` shows a SPIR model execution syntax

`-v` verbose

`-o` writes the output as Comma Separated Values (CSV) files

`-g` plots the output as a graphic

`configFile` identify the configuration file that specifies the parameters value to run the simulation (see Table S3)

`params` explicit specification of all parameters in the command-line through the symbols shown in Table S3

To execute the SPIR model from Linux terminal:

- Navigate to the `SPIR` directory
- Type

```
python SPIR/Main.py -v -g -o configFile config.txt
```

or

```
python SPIR/Main.py -v -g -o params -NS 9900 -NP 0 -NI 100 -NR
0 -PS 1 -PP 0.95 -PI 0.1 -PR 0.95 -BS 0.0303 -RH 0.1 -G 0.0152
-K 1 -D 0.0099 -H 90 -M 2 -R 10 -T 2100 -F 1 -W 100 -P ".." -N
"output" -O True -S ";"
```

A graphic with the dynamics of agents in the Susceptible, Prophylactic, Infectious, Recovered state in a panel and the proportion of infected agents over time in another are shown. In addition to generate the plot, output files will be generated with the raw data of the simulation.

S6.4 Configuration

You can configure the SPIR simulations by changing the values of the parameters in the `config.txt` file. Table S3 describes the configuration parameters for the SPIR model.

Table S3. Configuration parameters of the SPIR model.

Parameter		Description
Name	Symbol	
`num.agents.S`	-NS	Initial number of agents in state Susceptible
`num.agents.P`	-NP	Initial number of agents in state Prophylactic
`num.agents.I`	-NI	Initial number of agents in state Infectious
`num.agents.R`	-NR	Initial number of agents in state Recovered
`payoff.S`	-PS	Payoff received per time step in state Susceptible
`payoff.P`	-PP	Payoff received per time step in state Prophylactic
`payoff.I`	-PI	Payoff received per time step in state Infectious
`payoff.R`	-PR	Payoff received per time step in state Recovered
`beta`	-BS	Probability that an agent in state Susceptible becomes infected upon interacting with an Infectious agent
`rho`	-RH	Reduction in the transmission probability when adopting prophylactic behavior
`gamma`	-G	Probability an Infectious agent recover
`fear`	-K	Distortion of the perceived proportion of Infectious agents in the population (i.e. *distortion factor*)
`decision`	-D	Probability an agent in the Susceptible or Prophylactic state decides which behavior to engage in
`time.horizon`	-H	The time in the future over which agents calculate their utilities to make a behavioral decision
`method`	-M	Method of executing the simulation
`replication`	-R	Number of replications to run the scenario using different random seeds
`time.steps`	-T	Length of the simulation in steps
`output.format`	-F	Format of the output file (0 – Standard or 1 – Galapagos)
`output.window`	-W	Size of the window to consolidate the output of several replications
`output.path`	-P	Path of the output file
`output.file`	-N	Name of the output file without extension
`output.header`	-O	Flag that indicates whether or not to write the output's columns header in the output file
`output.separator`	-S	Character that separates the fields in the output file

S7 Comparison between the ABM and ODE Results

We implemented two versions of the SPIR model, an ABM and an ODE version. A drawback of the ABM version is that it requires simulations that can be computationally intensive. Because we assume in this paper that the population is well-mixed, we can generate the same dynamics using the ODE version, which is less computationally intensive.

Table S4. Input parameter values used in the comparison between ODE and ABM.

**Parameter**	**ODE**	**ABM**
N	100,000	100,000
Initial Susceptible agents	999,900	999,900
Initial Infectious agents	100	100
R_0	2	2
Recovery Time $(1/\gamma)$	65	65
β	0.0307	0.0303
ρ	0.01	0.0099
γ	0.0153	0.0152
δ	0.01	0.0099
κ	1	1
$\{u_S, u_P, u_I, u_R\}$	$\{1, 0.95, 0.1, 0.95\}$	$\{1, 0.95, 0.1, 0.95\}$

Figure S3 shows the results of a simulation with both models using the same input parameters listed in Table S4. Notice that the input parameter values differ because the ODE and ABM uses, respectively, rate and probability. We convert between rates and probabilities using equations $x = -\ln(1-y)$ and $y = 1 - e^{-x}$, where x and y are rate and probability values respectively [1]. One unit of continuous time in ODE corresponds to N time steps in ABM.

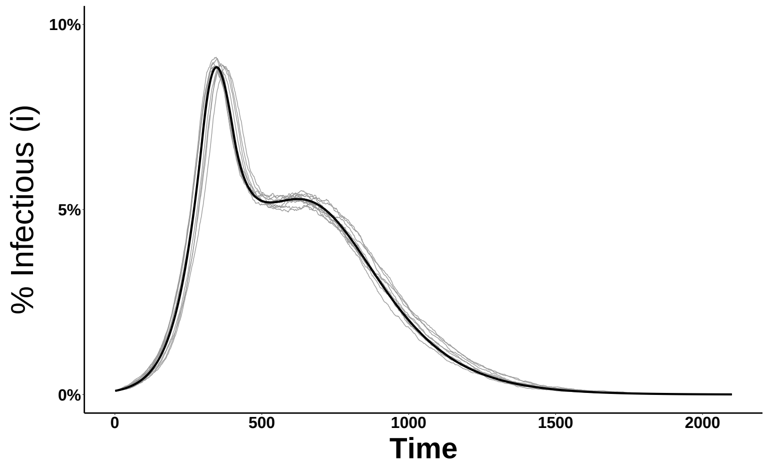

Figure S3. Proportion of infectious agents in the population over time.
The black line is the proportion of infectious agents generated using the ODE version. The gray lines are the proportion of infectious agents for the 10 replications generated using the ABM version.

References

[1] Rachael L. Fleurence and Christopher S. Hollenbeak. Rates and probabilities in economic modelling. *Pharmacoeconomics*, 25(1):3–6, 2007.
