# Supplementary figures and images for: Planning horizon affects prophylactic decision-making and epidemic dynamics
Source: PeerJ. 2016 Nov 8;4:e2678. doi: 10.7717/peerj.2678 (PMC5103819; doi:10.7717/peerj.2678)

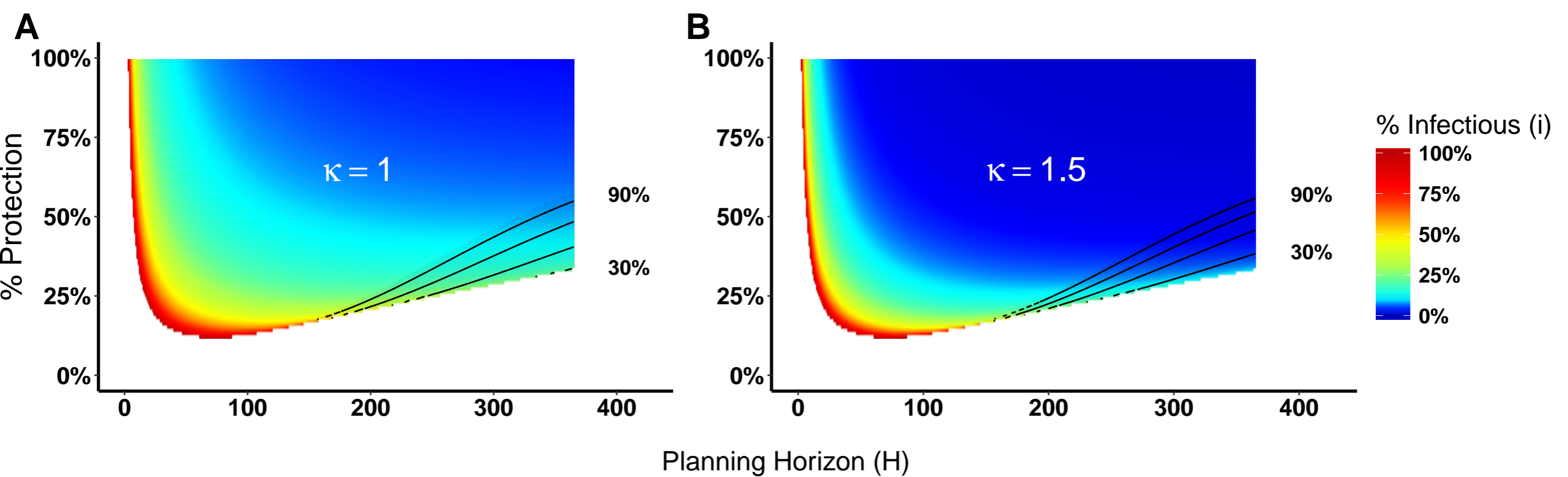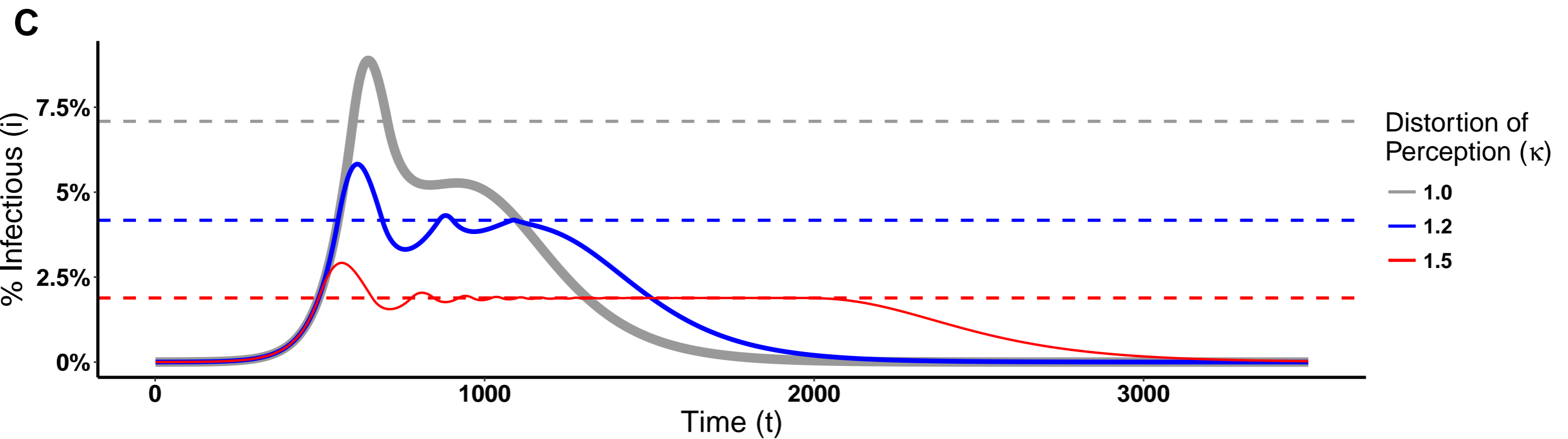

Supplement: Supplemental Information 1 [file peerj-04-2678-s001.zip › spir-paper-si/figures/figure7.pdf]

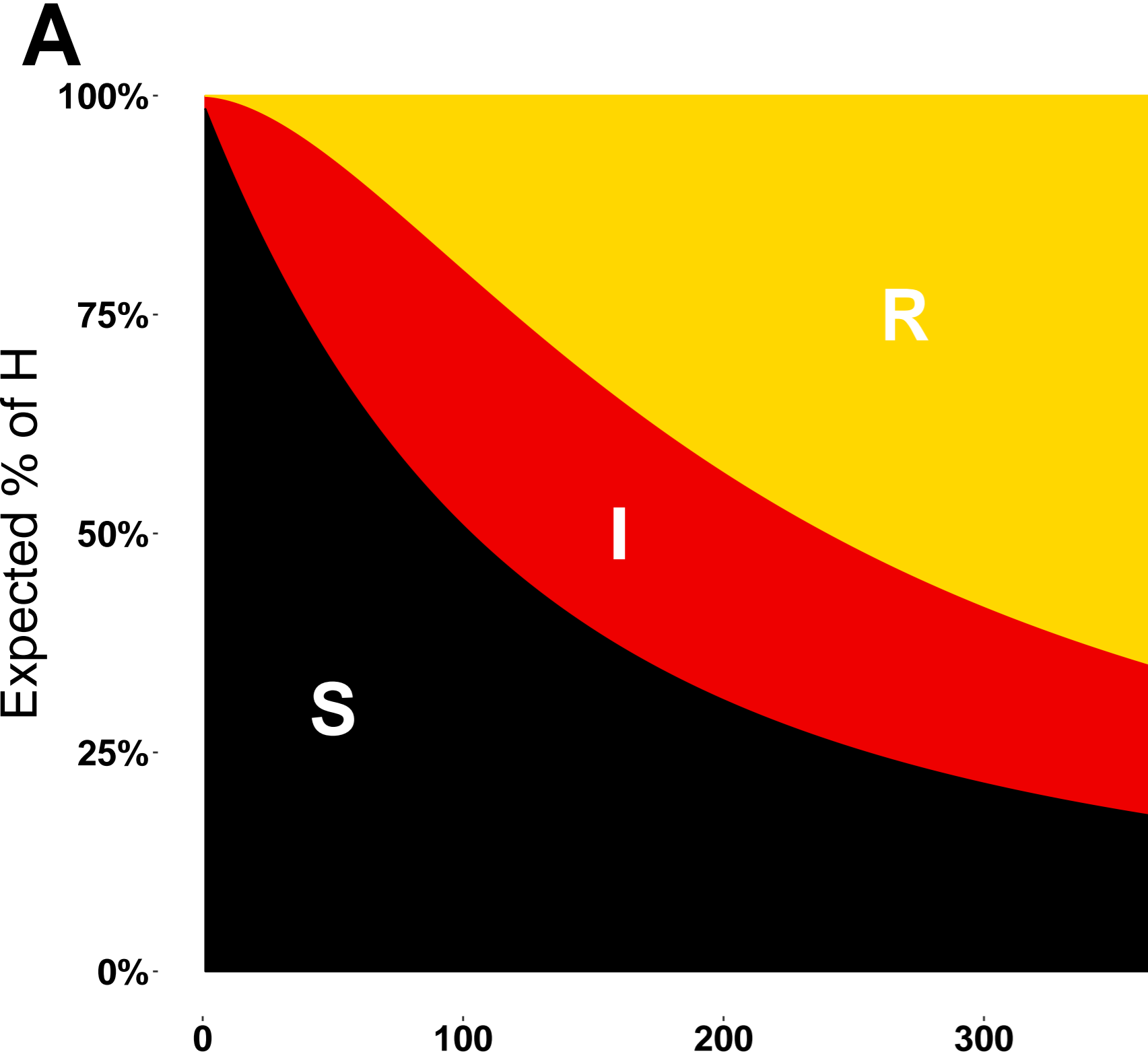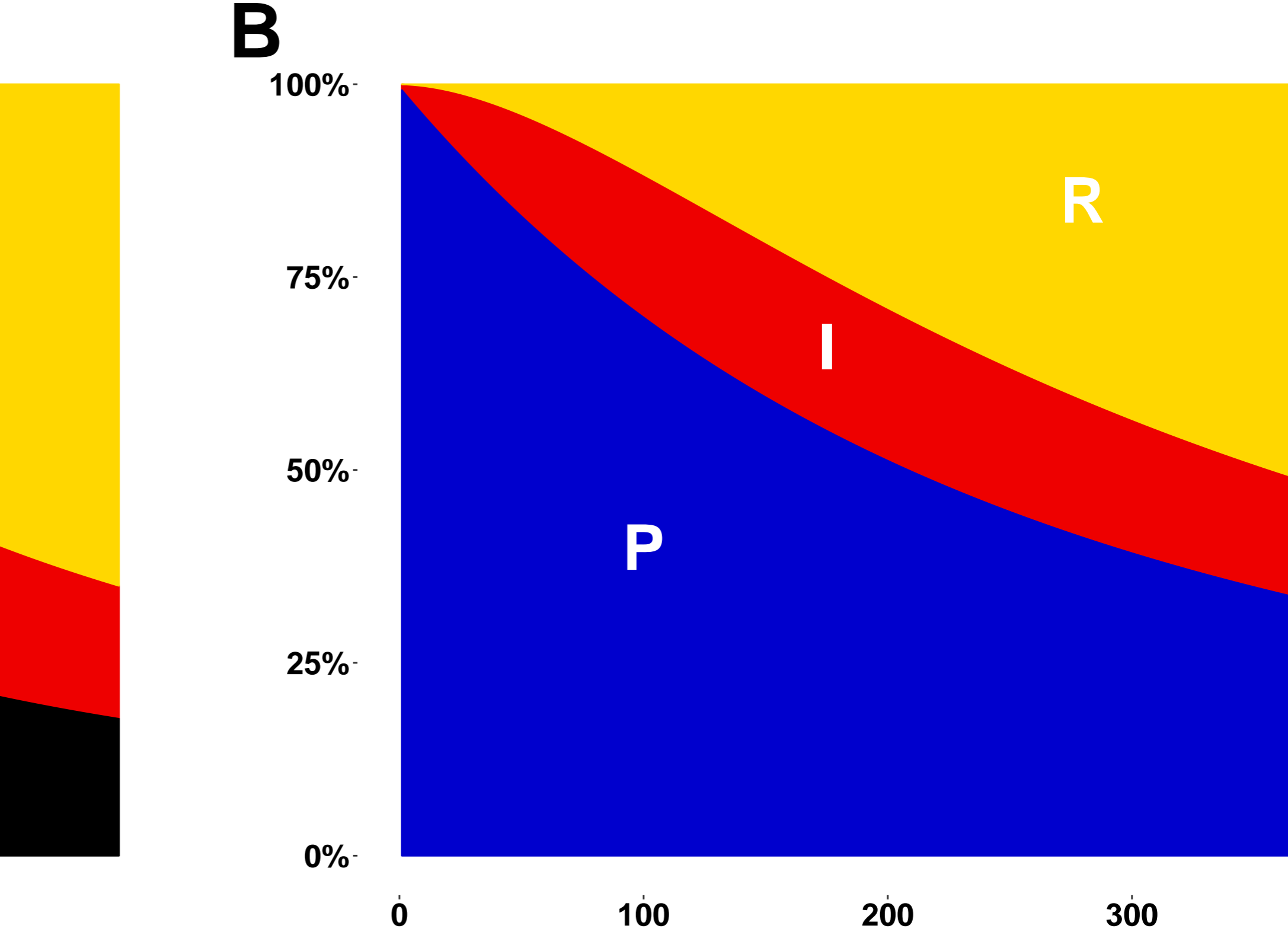

Planning Horizon (H)

Supplement: Supplemental Information 1 [file peerj-04-2678-s001.zip › spir-paper-si/figures/figure3.pdf]

% Infectious (i)

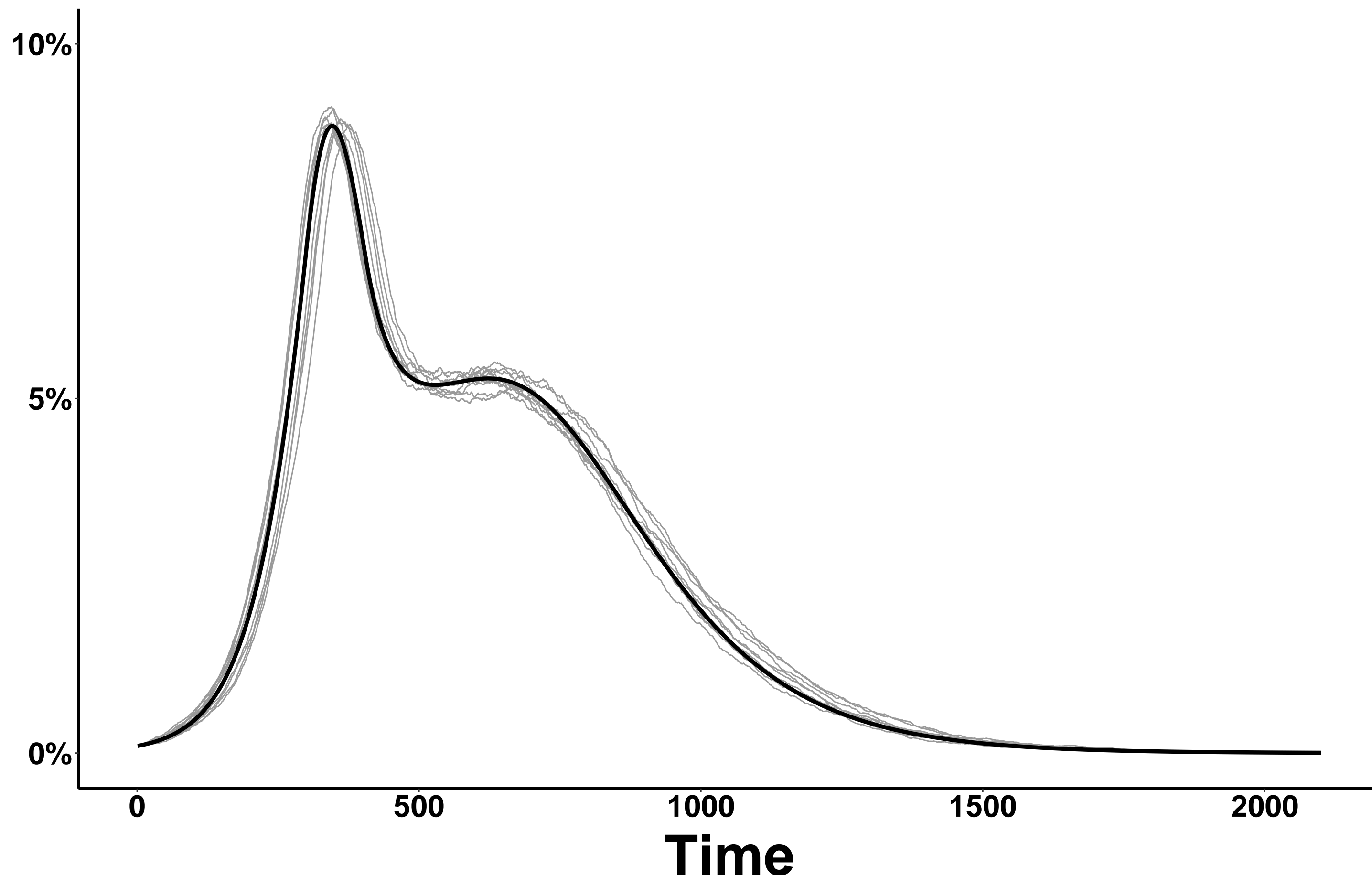

Supplement: Supplemental Information 1 [file peerj-04-2678-s001.zip › spir-paper-si/figures/figureS3.pdf]

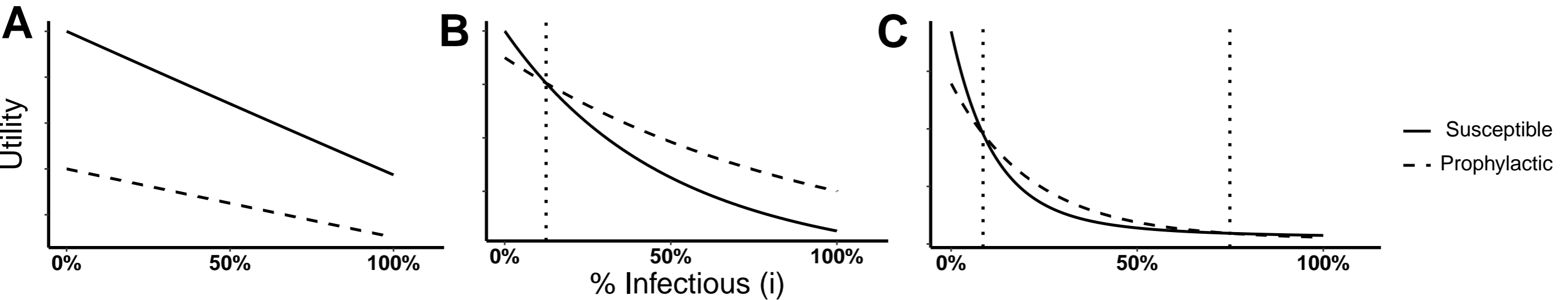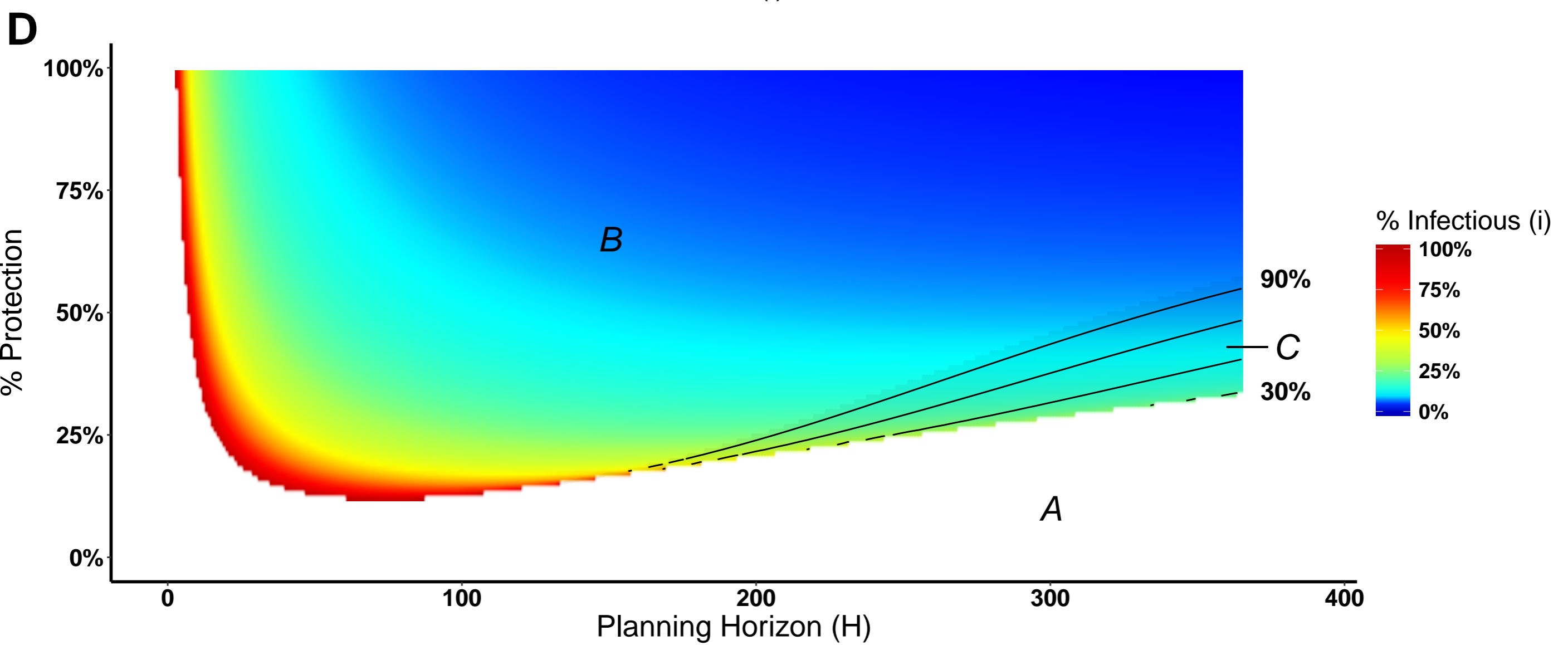

Supplement: Supplemental Information 1 [file peerj-04-2678-s001.zip › spir-paper-si/figures/figure2.pdf]

**A**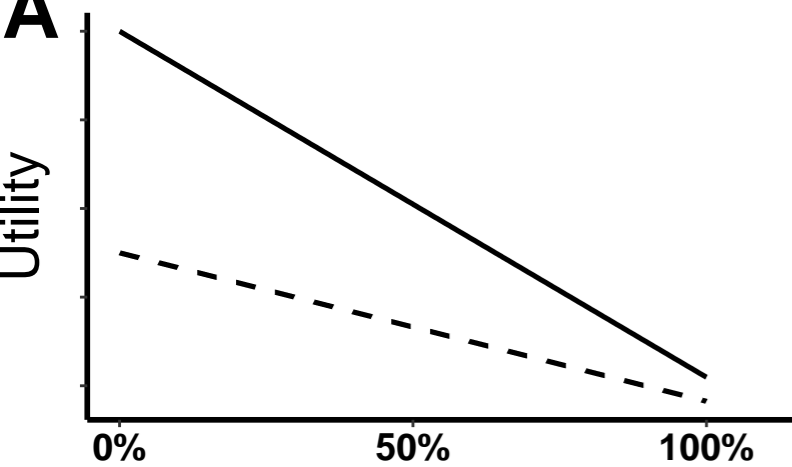**B**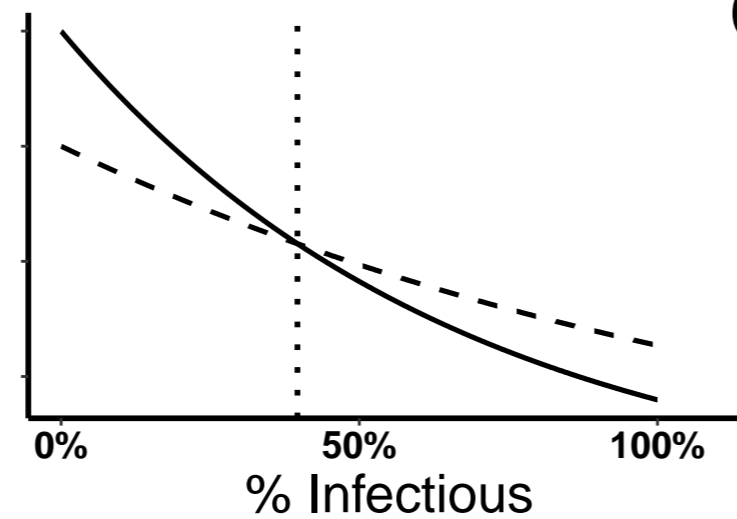**C**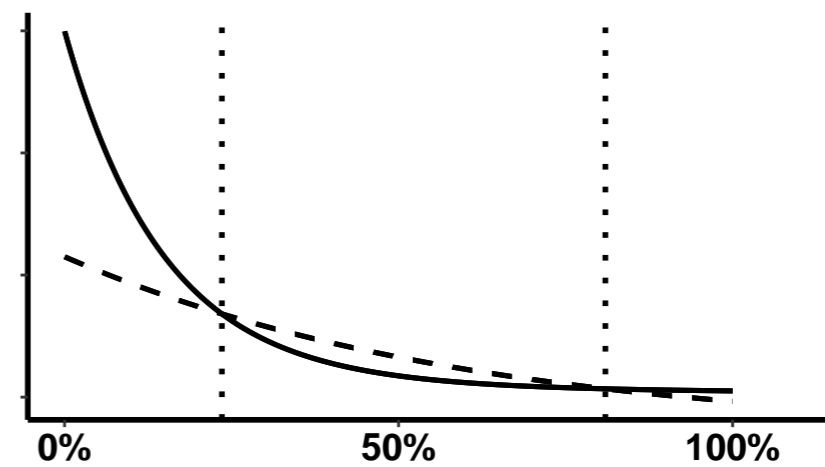

— Susceptible  
- - Prophylactic

**D**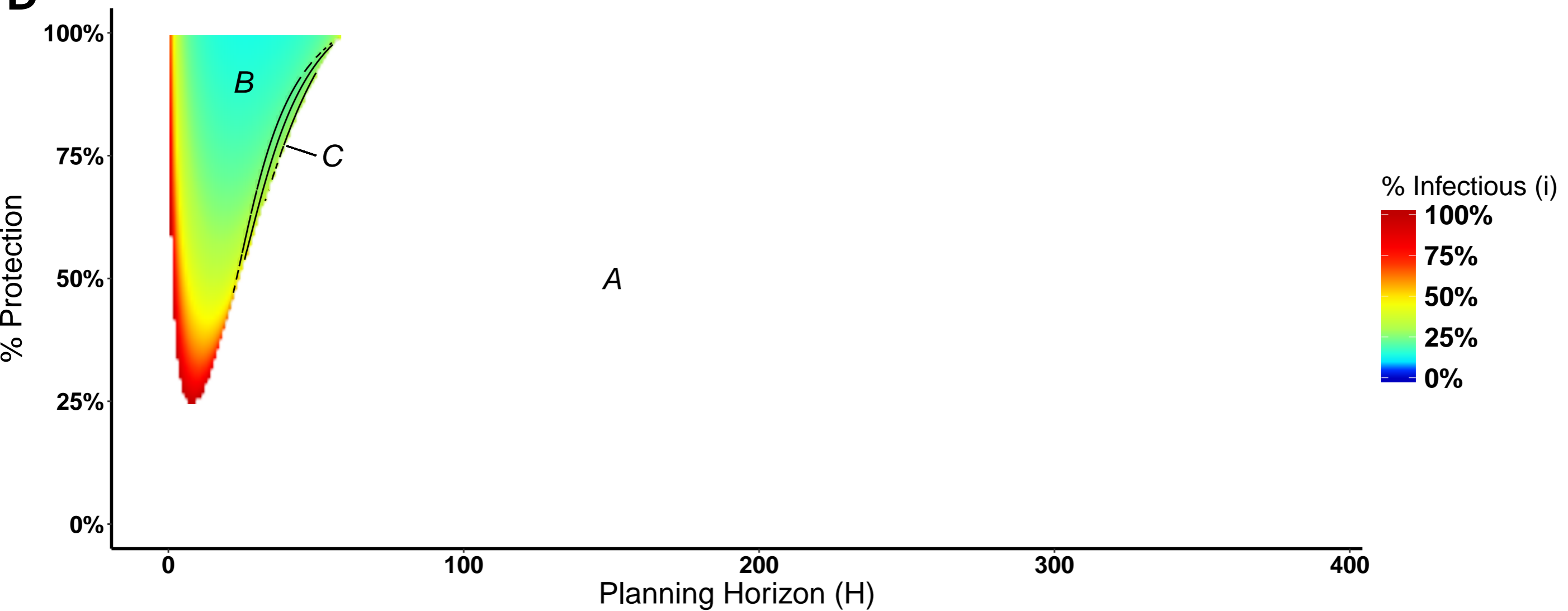

Supplement: Supplemental Information 1 [file peerj-04-2678-s001.zip › spir-paper-si/figures/figureS1.pdf]

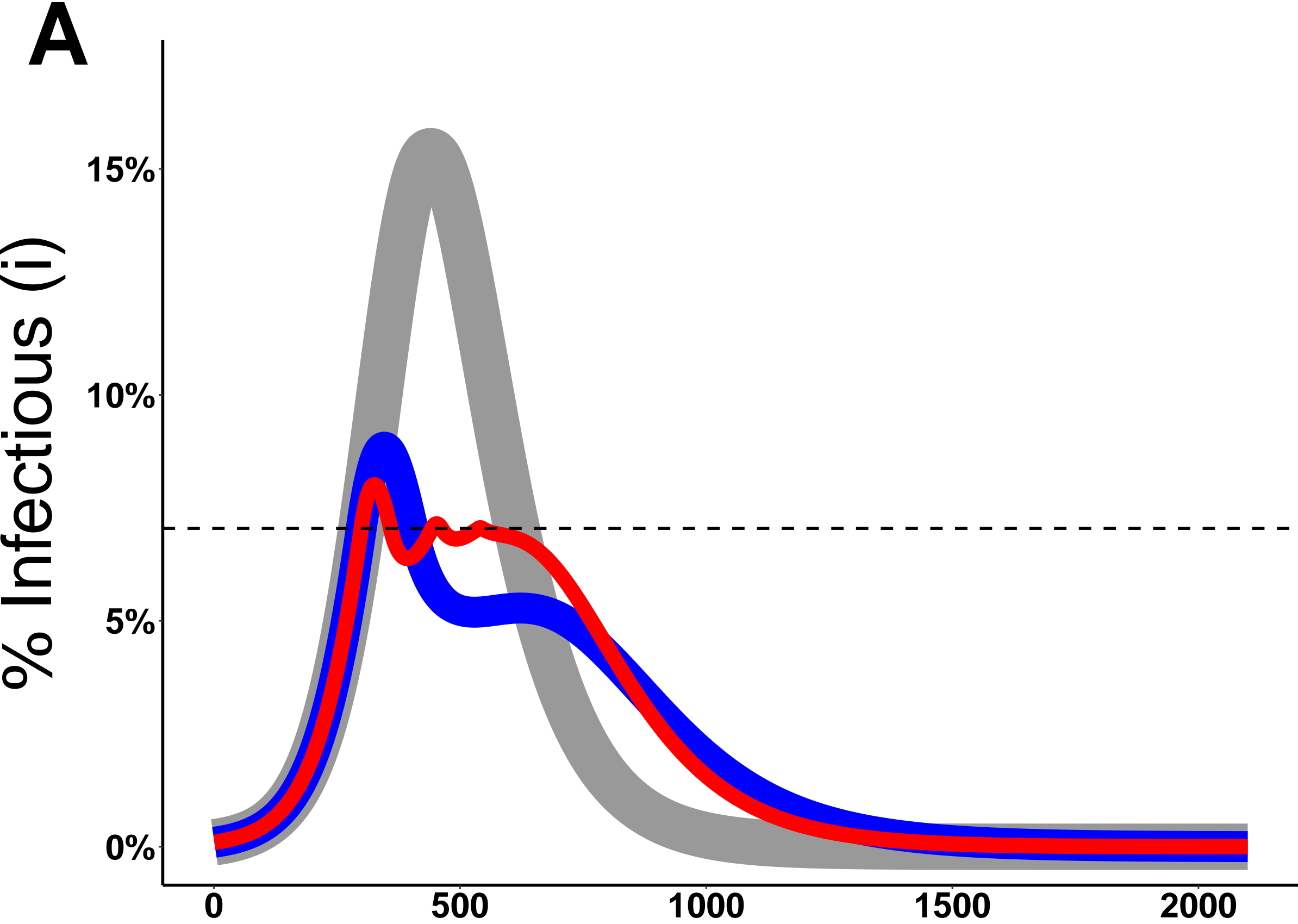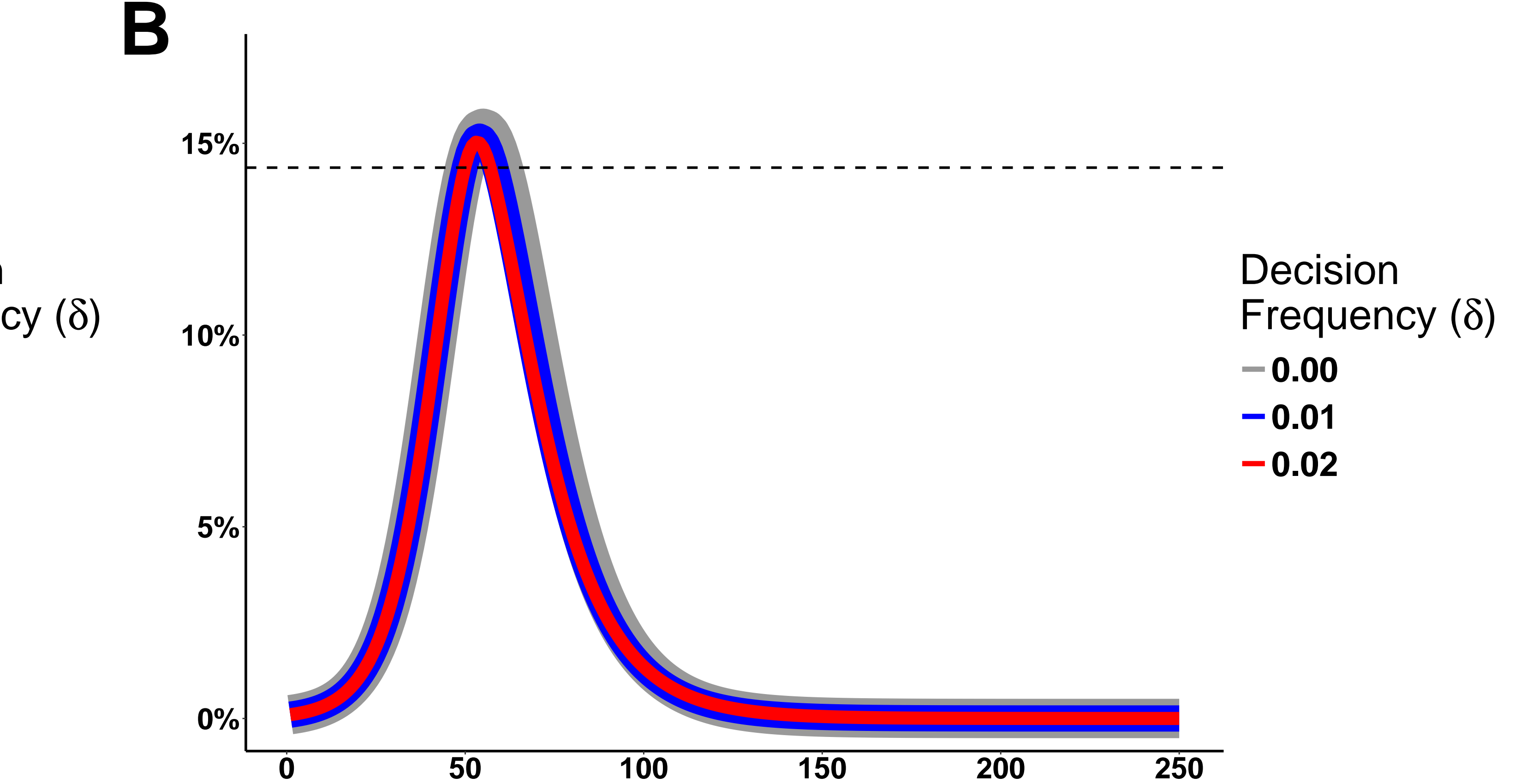

Time (t)

Supplement: Supplemental Information 1 [file peerj-04-2678-s001.zip › spir-paper-si/figures/figure6.pdf]

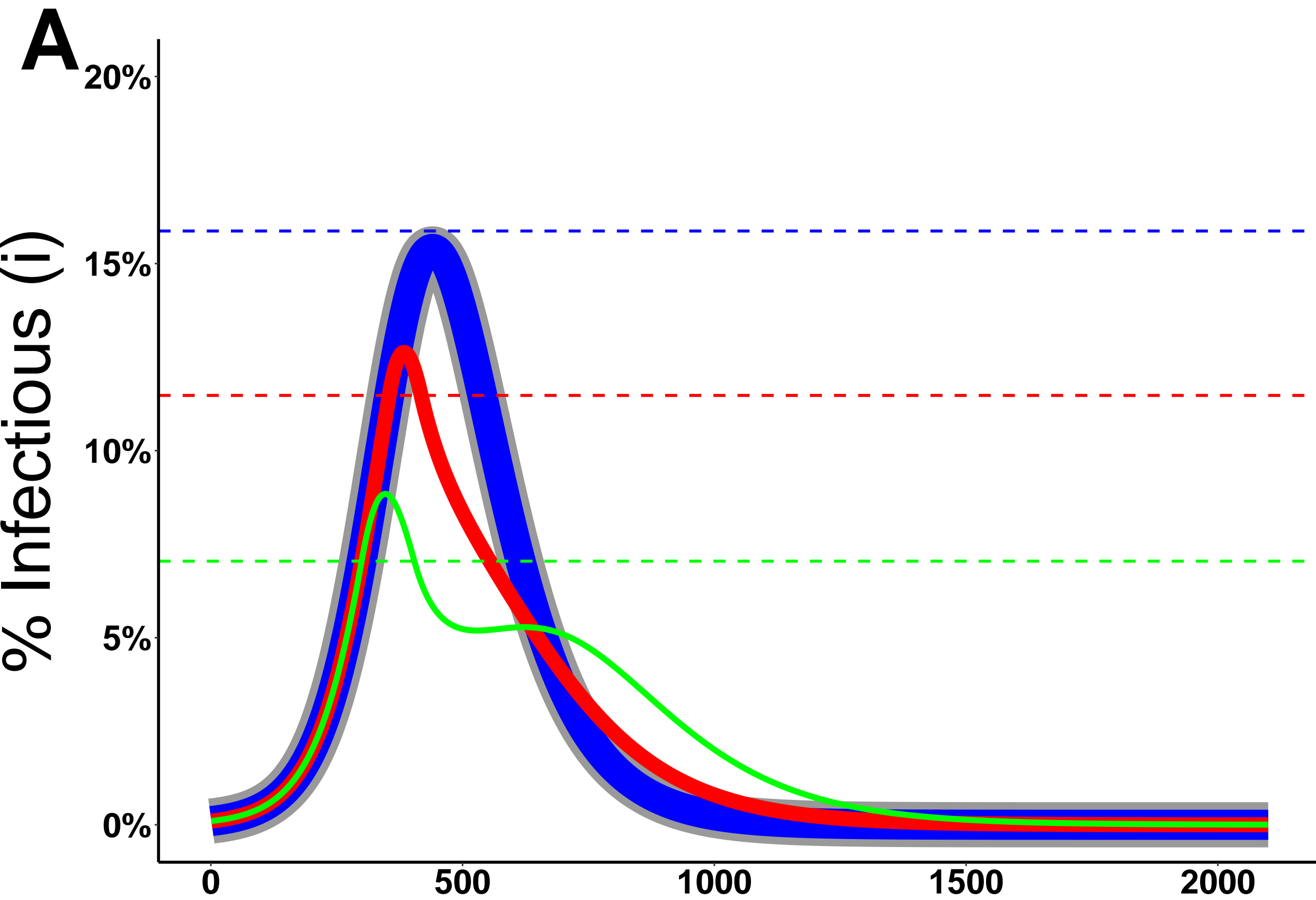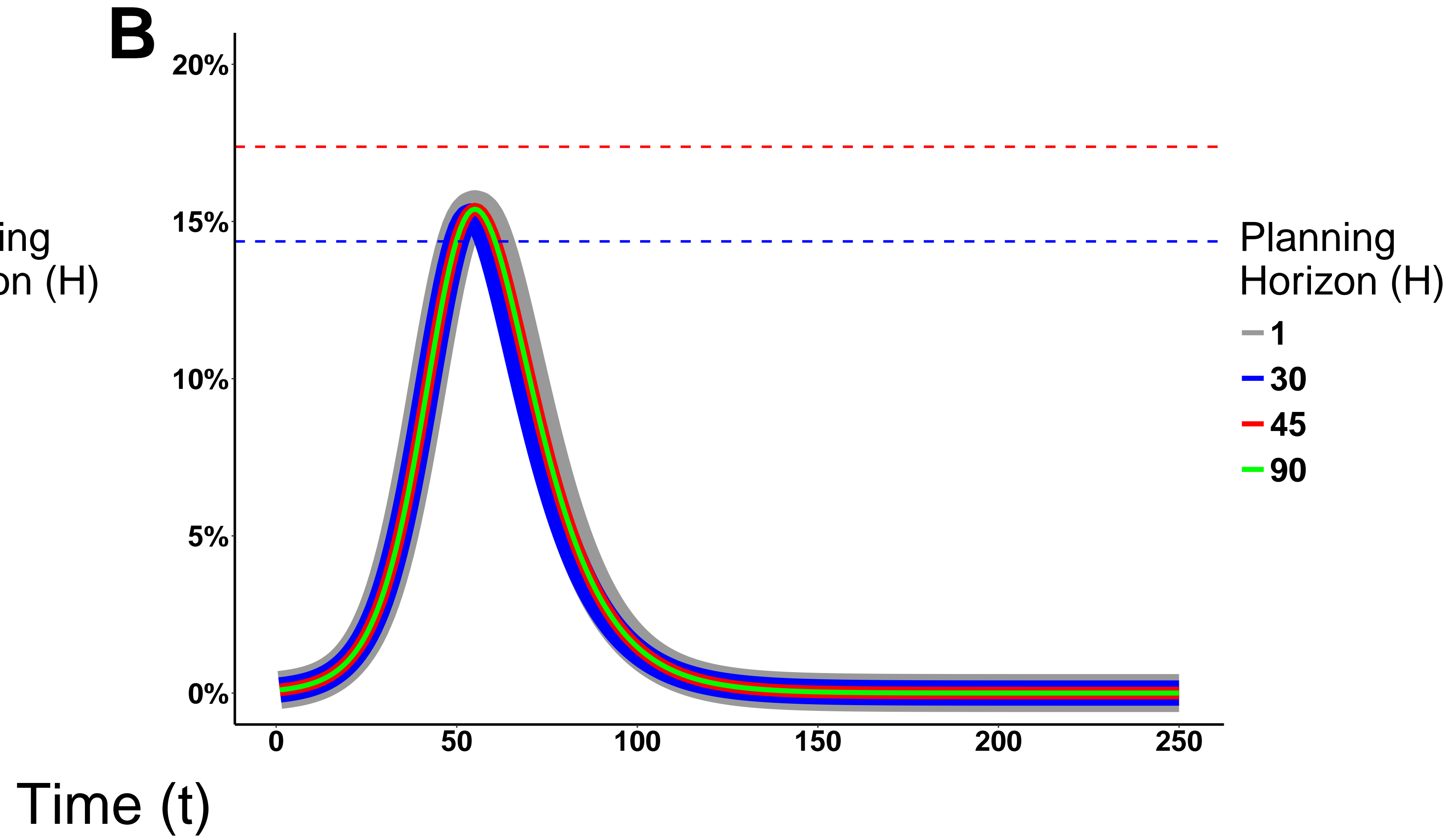

Supplement: Supplemental Information 1 [file peerj-04-2678-s001.zip › spir-paper-si/figures/figure5.pdf]

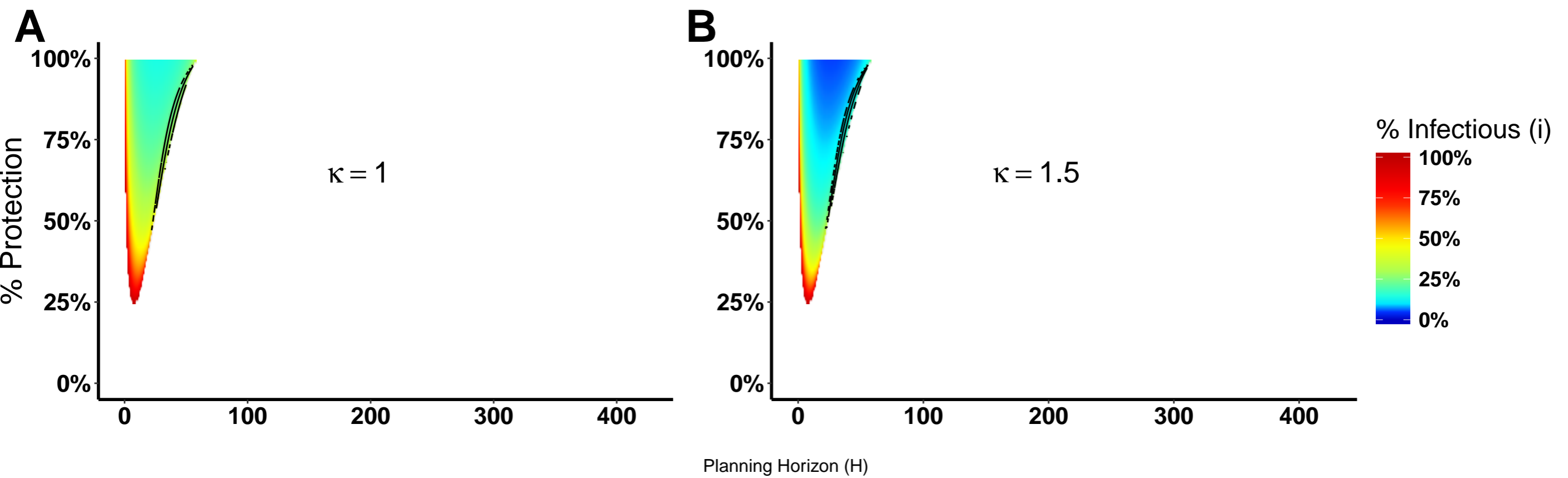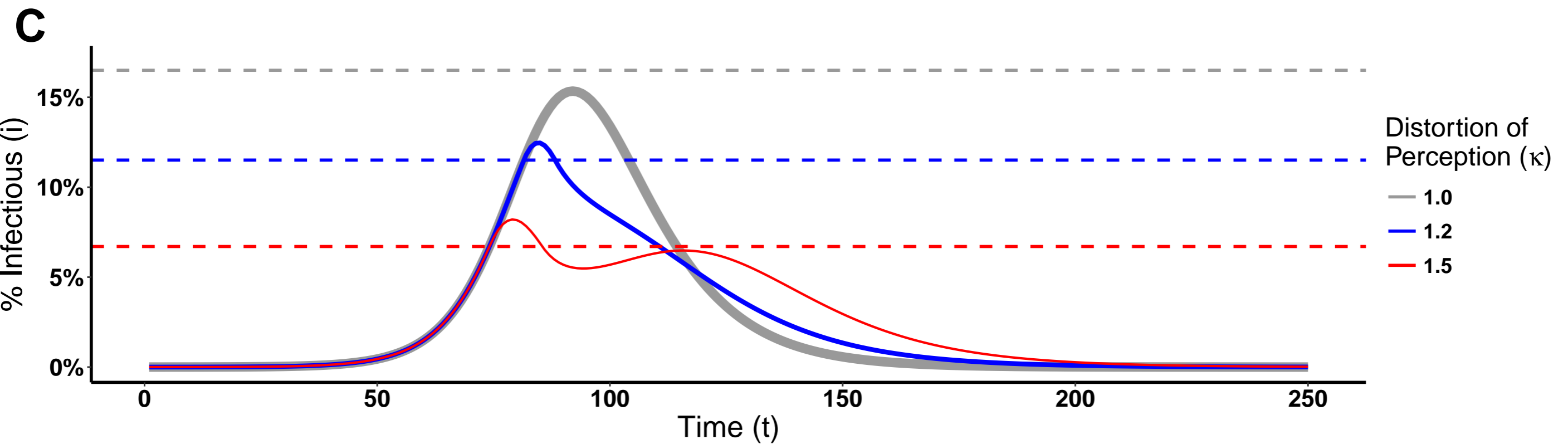

Supplement: Supplemental Information 1 [file peerj-04-2678-s001.zip › spir-paper-si/figures/figureS2.pdf]
